# Synthesis, Characterization and Application of NNN Pincer Manganese Complexes with Pyrazole Framework in α-Alkylation Reaction

**DOI:** 10.3390/molecules30071465

**Published:** 2025-03-26

**Authors:** Tao Wang, Yongli Xu, Mengxin Du, Zhiyuan Hu, Lantao Liu

**Affiliations:** 1 Henan Engineering Laboratory of Green Synthesis for Pharmaceuticals, School of Chemistry and Chemical Engineering, Shangqiu Normal University, Shangqiu 476000, China; 2 College of Petrochemical Engineering, Liaoning Petrochemical University, Fushun 113001, China

**Keywords:** NNN pincer manganese complexes, pyrazole skeleton, α-alkylation reaction

## Abstract

A series of novel NNN pincer manganese complexes based on pyrazole skeleton **4** were efficiently synthesized in a two-step process. All of the new complexes were fully characterized by ^1^H, ^13^C NMR spectra. Furthermore, the molecular structures of complexes **4a** and **4c** were also determined by X-ray single-crystal diffraction. The manganese(I) catalysts obtained showed efficient catalytic activity in the α-alkylation reaction of ketones with alcohols. Under optimal reaction conditions, the expected products were procured with moderate to high yields.

## 1. Introduction

**Scheme 1:**
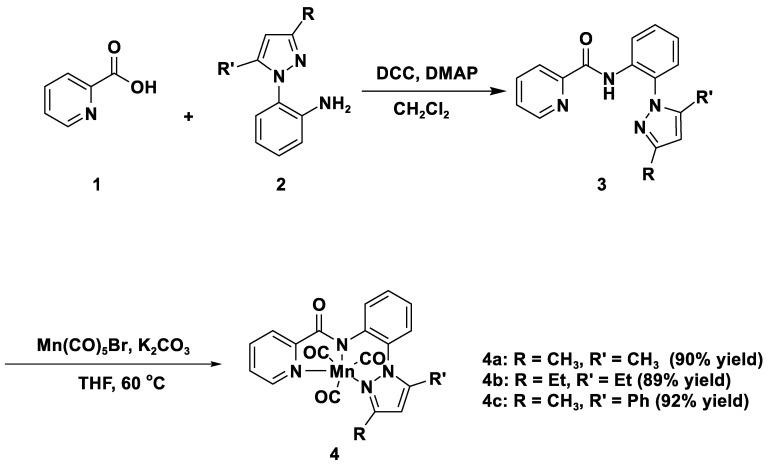
Synthesis of the NNN pincer manganese complexes based on pyrazole skeleton.

Over the past few decades, the synthesis of pincer complexes and their applications in catalysis have been significant research topics in the field of organometallic chemistry [1,2,3,4,5,6,7,8]. To date, a wide array of pincer complexes, including those of palladium [9,10,11,12,13], ruthenium [14,15,16,17,18,19], copper [20,21,22], nickel [23,24,25], cobalt [26,27,28,29,30], and zinc [31,32], have been successfully synthesized and utilized as catalysts in numerous challenging chemical reactions. Despite these advancements, the design, synthesis, and application of pincer manganese complexes remain in their infancy. In fact, after iron and titanium, manganese is the third most abundant transition metal in the Earth’s crust. Since the first report of a manganese pincer catalyst in 2016 by the group of Milstein [33], pincer manganese complexes have garnered the attention of many scientists. For instance, the corresponding PNP-manganese pincer complexes [34,35,36,37,38] have been applied to the Guerbet reaction of ethanol, C-alkylation of secondary alcohols, dehydrogenative coupling, and conjugate addition reactions. A novel type of PNN pincer ligand and its corresponding manganese complexes [39,40,41,42,43] have been developed and further applied in the transfer hydrogenation of esters, and hydrogenation reactions, as well as N-alkylation of amines. Due to the well-documented air and moisture sensitivities and the high cost of phosphine ligands, the development of phosphine-free pincer manganese complexes has been reported successively [44,45,46,47]. Despite considerable progress, the construction of new functional organic compounds catalyzed by known metal pincer complexes, as well as the synthesis and applications of new pincer complexes, will continue to be significant contributions to the field of pincer chemistry. Recently, we reported a simplified method for synthesizing pincer palladium complexes [48,49,50] and NHC-palladium complexes [51,52,53,54] featuring tridentate N-heterocyclic ligands. The palladium complexes obtained exhibited outstanding catalytic activity in various catalytic reactions. To expand the scope of pincer metal chemistry and in continuation of our investigations into the construction of functionalized complexes [55,56], we would like to describe the NNN pincer manganese complexes based on a pyrazole skeleton, which can also be conveniently synthesized using 2-pyridinecarboxylic acid and 2-pyrazolyl anilines as starting materials (Scheme 1).

## 2. Results and Discussion

### 2.1. Synthesis and Characterization of the Complexes

The synthesis of the required ligands **3** was easily carried out from commercially available 2-pyridinecarboxylic acid, as shown in Scheme 1. With the ligands in hand, the coordination was carried out by heating the ligands **3a**–**c** with Mn(CO)_5_Br in toluene in the presence of K_2_CO_3_ to afford the air- and moisture-stable pincer manganese complexes **4a**–**c** in good yields. All of the new complexes were fully characterized by ^1^H NMR and ^13^C NMR. Additionally, the X-ray crystal structures of complex **4a** and **4c** were obtained.

**Figure 1:**
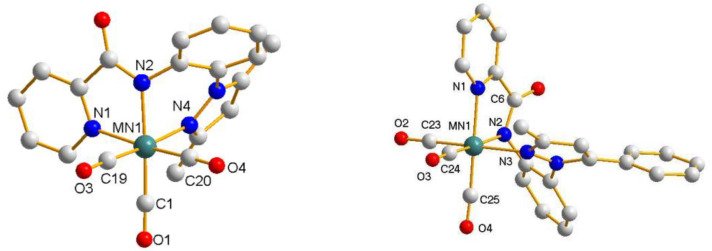
Molecular structures of the Mn(I) complexes **4a** (**left**) and **4c** (**right**). Hydrogen atoms are omitted for clarity.

The molecular structures of pincer manganese complexes **4a** and **4c** were unambiguously determined by X-ray single-crystal analysis. The molecules are illustrated in Figure 1. The bond lengths and bond angles selected are listed in Table 1. The molecular structure of each complex exhibits an octahedral geometry with the pincer ligand coordinated to the metal via the pendant pyridine-N, amide-N, and pyrazol-N in tridentate manner and three carbonyl ligands occupying the other coordination sites. Complexes **4a** and **4c** possess one five-membered and one six-membered manganese ring. All of the bond lengths and angles around the Mn(I) center in the two complexes are similar, and the N(amide)-Mn-C angles (174°) are almost linear. The values of bond lengths and angles are comparable to those in the related pincer manganese complexes [57]. In complex **4a**, the Mn-N bond lengths are 2.074(4) Å, 2.003(3) Å and 2.076(4) Å, respectively. The N(1)-Mn(1)-N(2), N(1)-Mn(1)-N(4) and N(2)-Mn(1)-N(4) angles in this complex are 77.65(14)°, 92.83° and 80.10(14)°, respectively. Complex **4c** has very similar bond lengths and angles around the Mn(I) center, although it contains a different substituent on its pyrazol ring from **4a**.

### 2.2. Catalytic Studies

The α-alkylation reaction of carbonyl compounds offers a valuable method for constructing a range of functionalized organic molecules, which exhibit activities in pharmaceuticals, materials science, and agricultural chemicals [19]. Recently, several research groups have achieved favorable outcomes in α-alkylation reactions by employing synthetic cobalt [58], ruthenium [59,60], and zinc [61] as catalysts. Furthermore, an excess of base can also facilitate the occurrence of the α-alkylation reaction [62]. However, the aforementioned literature reports still present numerous issues, such as complex catalyst synthesis processes and excessive amounts of alkali used. Therefore, it is imperative to develop more effective strategies for the α-alkylation reaction. In this paper, the α-alkylation reaction of acetophenone **5a** with 4-methoxybenzyl alcohol **6a**, using the current NNN pincer manganese complex **4c** as a catalyst and KO^t^Bu as the base, was conducted in toluene at 140 °C for 14 h. To our satisfaction, the desired product **7aa** was produced with a 35% yield (Table 2, entry 1). Various other bases, including KOH, K_2_CO_3_, Na_2_CO_3_, NaO^t^Bu, NaOH, LiCO_3_, LiO^t^Bu, Cs_2_CO_3_, and Et_3_N, were subsequently tested (Table 3, entries 2–10). Notably, NaOH emerged as the superior base, yielding the product at 81% (entry 6). This underscores the importance of the base selection on the reaction’s yield [63]. After exploring various solvents, including 1,4-Dioxane, m-xylene, tert-amyl alcohol, THF, and DMF (entries 11–15), it was found that none of these solvents improved the product yield. Finally, the effectiveness of two other NNN pincer manganese complexes **4a**–**4b** under these reaction conditions was evaluated, and complex **4c** was determined to be the most effective (Table 2, entries 16–17 compared to entry 6). The product yield was determined to be 57% under reaction conditions with the temperature set at 120 °C (entry 18). When 2.0 mol% of complex **4c** was tested, the yield of the product decreased significantly (entry 19).

With the optimized conditions at our disposal, we began to systematically explore the generality of this methodology. Various alcohols were tested for alkylation with **5a**. As presented in Table 3, the majority of the α-alkylation reactions proceeded efficiently, yielding the corresponding products **7aa**–**7ah** with good to excellent results. Both electron-donating and electron-withdrawing groups on the phenyl ring of the aryl alcohols were tolerated (**7aa**–**7af**). Generally, the presence of electron-donating groups in the phenyl ring of aryl alcohols had a beneficial effect on the yields of the catalytic products. However, the presence of substituents at the ortho or para positions of benzyl alcohol could lead to a reduction in product yields (**7ab** and **7ac**). We attempted to use 4-nitrobenzyl alcohol and 4-trifluoromethylbenzyl alcohol as substrates for the reaction, but, unfortunately, the target compound was not obtained. Additionally, substrate **6**, which contains a naphthalene ring substituent, was also suitable for such transformation, producing products **7ag** and **7ah** in good yields under appropriate conditions.

Subsequently, we examined the scope of the ketone component (Table 4). A broad spectrum of ketones incorporating diverse functional groups were well tolerated, resulting in the desired products **7ba**–**7ha** with good yields. Overall, the current findings indicated that the NNN pincer manganese complex **4c** remained efficient in the α-alkylation reaction process.

## 3. Experimental Section

### 3.1. General

The preparation of the NNN pincer manganese complexes and all catalytic reactions were conducted under a nitrogen atmosphere. Ligand **3** was synthesized following the procedure described in the literature [48]. Solvents were dried using standard methods and distilled freshly before use. All other chemicals were used as purchased. Melting points were determined using an XT4A melting point apparatus and are uncorrected. ^1^H and ^13^C NMR spectra were obtained on a Germany Bruker DPX 400 instrument, with TMS serving as the internal standard.

### 3.2. General Procedure for Synthesis of the NNN Pincer Manganese(I) Complexes 4

To a stirred solution of **3** (0.20 mmol) and K_2_CO_3_ (0.30 mmol) in THF (2 mL), Mn(CO)_5_Br (0.30 mmol) was added under a N_2_ atmosphere, and the reaction mixture was reacted for 12 h. After cooling, filtration, and evaporation, the residue was purified by preparative TLC on silica gel plates to afford the corresponding NNN pincer manganese(I) complexes **4a**–**c**.

**Complex** (**4a**): 77.4 mg, 90% yield. Yellow solid. mp: 220–223 °C. ^1^H NMR (400 MHz, CDCl_3_) δ 9.02 (d, *J* = 3.8 Hz, 1H), 8.05 (d, *J* = 6.4 Hz, 1H), 7.90 (s, br, 1H), 7.65 (d, *J* = 6.4 Hz, 1H), 7.46 (d, *J* = 5.9 Hz, 2H), 7.26 (s, br, 2H), 5.95 (s, 1H), 2.36 (s, br, 6H). ^13^C NMR (100 MHz, CDCl_3_): δ 166.7, 157.3, 152.7, 151.9, 146.8, 142.9, 138.5, 133.1, 128.6, 126.1, 125.8, 125.2, 123.8, 123.5, 109.8, 15.2, 14.5.

**Complex** (**4b**): 81.5 mg, 89% yield. Yellow solid. mp: 228–230 °C. ^1^H NMR (400 MHz, CDCl_3_) δ 9.00 (d, *J* = 5.1 Hz, 1H), 8.04 (d, *J* = 7.2 Hz, 1H), 7.89 (t, *J* = 7.2 Hz, 1H), 7.65 (d, *J* = 7.5 Hz, 1H), 7.48–7.43 (m, 2H), 7.28 (d, *J* = 7.6 Hz, 1H), 7.21 (t, *J* = 7.6 Hz, 1H), 6.04 (s, 1H), 3.08–2.98 (m, 1H), 2.88–2.80 (m, 1H), 2.66–2.57 (m, 2H), 1.18 (t, *J* = 7.4 Hz, 3H), 1.08 (t, *J* = 7.5 Hz, 3H). ^13^C NMR (100 MHz, CDCl_3_): δ 166.4, 157.8, 157.4, 152.7, 149.4, 147.1, 138.4, 133.1, 128.5, 126.0, 125.9, 125.1, 123.7, 123.6, 105.5, 22.5, 21.6, 13.4, 12.7.

**Complex (4c)**: 90.5 mg, 92% yield. Yellow solid. mp: 234–236 °C. ^1^H NMR (400 MHz, CDCl_3_) δ 9.05 (s, 1H), 8.08 (s, br, 1H), 7.92 (d, br, 1H), 7.63 (d, *J* = 6.0 Hz, 1H), 7.49 (s, br, 1H), 7.35–7.26 (m, 4H), 7.20 (s, 2H), 6.86 (s, br, 1H), 6.66 (d, *J* = 6.8 Hz, 1H), 6.17 (s, 1H), 2.48 (s, 3H). ^13^C NMR (100 MHz, CDCl_3_): δ 153.1, 152.8, 147.6, 146.9, 138.7, 133.2, 130.1, 129.2, 129.1, 128.7, 128.5, 126.1, 126.0, 125.9, 125.4, 123.7, 109.9, 15.6.

### 3.3. General Procedure for the α-Alkylation Reaction

A Schlenk flask was charged with the required ketone (0.50 mmol), alcohol (0.75 mmol), NNN pincer manganese(I) complex (5.0 mol %), NaOH (50.0 mol %), and toluene (2.0 mL). The mixture was stirred at 140 °C for 14 h under N_2_. After cooling, the reaction mixture was evaporated and the product was isolated by preparative TLC on silica gel plates. The purified products were identified by NMR spectra and their analytical data are given in the Supporting Materials.

### 3.4. Crystal Structure Determination and Data Collection

Crystals of complex **4a** (CCDC 2422192) and **4c** (CCDC 2422233) were obtained by recrystallization from CH_2_Cl_2_/*n*-hexane at ambient temperature. Data were collected on an Oxford Diffraction Gemini E diffractometer with graphite-monochromated Mo Kα radiation (λ = 0.7107 Å). The structure was solved by direct methods using the SHELXS-97 program, and all non-hydrogen atoms were refined anisotropically on *F*^2^ by the full-matrix least-squares technique, using the SHELXL-97 crystallographic software package [64,65]. The hydrogen atoms were included but not refined. Details of the crystal structure determination are summarized in Table 5. The crystallographic data can be obtained free of charge from The Cambridge Crystallographic Data Centre via http://www.ccdc.cam.ac.uk/data_request/cif (accessed on 7 December 2024).

## 4. Conclusions

In conclusion, a new series of phosphine-free pincer manganese complexes based on a pyrazole framework have been synthesized and utilized as catalysts for the α-alkylation of ketones with alcohols. Ongoing research in our laboratory focuses on further modifications of these NNN pincer manganese complexes and their application as catalysts in various other reactions.

## Figures and Tables

**Table 1:** Selected bond lengths (Å) and angles (deg) for complexes **4a** and **4c**.

	4a	4c^.^H_2_O
Mn1-N1	2.074(4) Å	2.081(5) Å
Mn1-N2	2.003(3) Å	2.025(5) Å
Mn1-N4/Mn1-N3	2.076(4) Å	2.101(5) Å
Mn1-C1/Mn1-C24	1.806(5) Å	1.805(7) Å
Mn1-C19/Mn1-C23	1.804(5) Å	1.792(9) Å
Mn1-C20/Mn1-C25	1.808(5) Å	1.801(7) Å
C1-Mn1-N2/C24-Mn1-N2	174.0°	174.4°
N(1)-Mn1-N4/N(1)-Mn1-N3	92.83°	91.3°
C19-Mn1-C1/C23-Mn1-C24	87.6(2)°	89.8(3)°
C1-Mn1-C20/C24-Mn1-C25	88.6(2)°	88.6(3)°
C19-Mn1-N2/C23-Mn1-N2	94.92(18)°	94.1(3)°
C20-Mn1-N2/C25-Mn1-N2	96.82(19)°	95.6(3)°
C1-Mn1-N1/C24-Mn1-N1	97.1(2)°	98.1(3)°
N2-Mn1-N1	77.65(14)°	78.0(2)°
C1-Mn1-N4/C24-Mn1-N3	97.6(2)°	94.7(3)°
N2-Mn1-N4/N2-Mn1-N3	80.10(14)°	81.5(2)°

**Table 2:** Optimization of reaction conditions for α-alkylation reaction of acetophenone with 4-methoxybenzyl alcohol catalyzed by the NNN-Mn(I) complexes *^a^*.

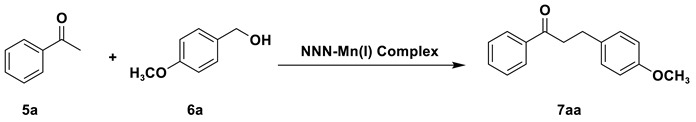				
**Entry**	**Cat.**	**Base**	**Solvent**	**Yield (%) *^b^***
1	**4c**	KO*^t^*Bu	Toluene	35
2	**4c**	KOH	Toluene	37
3	**4c**	K_2_CO_3_	Toluene	NR
4	**4c**	Na_2_CO_3_	Toluene	NR
5	**4c**	NaO*^t^*Bu	Toluene	75
6	**4c**	NaOH	Toluene	81
7	**4c**	Li_2_CO_3_	Toluene	NR
8	**4c**	LiO*^t^*Bu	Toluene	11
9	**4c**	Cs_2_CO_3_	Toluene	26
10	**4c**	Et_3_N	Toluene	NR
11	**4c**	NaOH	1,4-Dioxane	37
12	**4c**	NaOH	m-xylene	60
13	**4c**	NaOH	tert-amyl alcohol	40
14	**4c**	NaOH	THF	28
15	**4c**	NaOH	DMF	NR
16	**4a**	NaOH	Toluene	65
17	**4b**	NaOH	Toluene	63
18 *^c^*	**4c**	NaOH	Toluene	57
19 *^d^*	**4c**	NaOH	Toluene	37

*^a^* All reactions were carried out using **5a** (0.50 mmol), **6a** (0.75 mmol), base (50 mol%), catalyst (5.0 mol%) in solvent (2.0 mL) at 140 °C for 14 h. *^b^* Isolated yields. *^c^* at 120 °C. *^d^* Catalyst (2.0 mol%).

**Table 3:** Scope of alcohol substrates *^a^^,b^*.

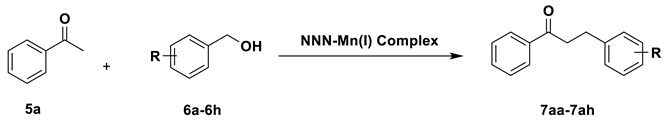
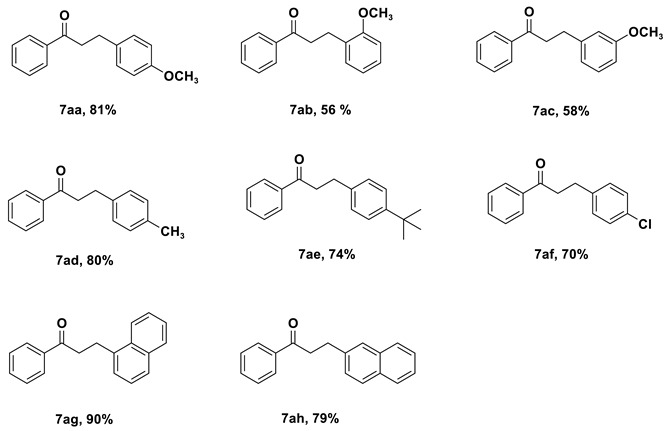

*^a^* All reactions were carried out using acetophenone (0.50 mmol), alcohol (0.75 mmol), NaOH (50 mol%), plus catalyst **4c** (5.0 mol%) in toluene (2.0 mL) at 140 °C for 14 h. *^b^* Isolated yields.

**Table 4:** Scope of ketone substrates *^a^^,b^*.

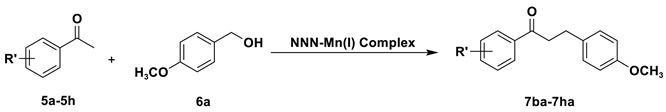
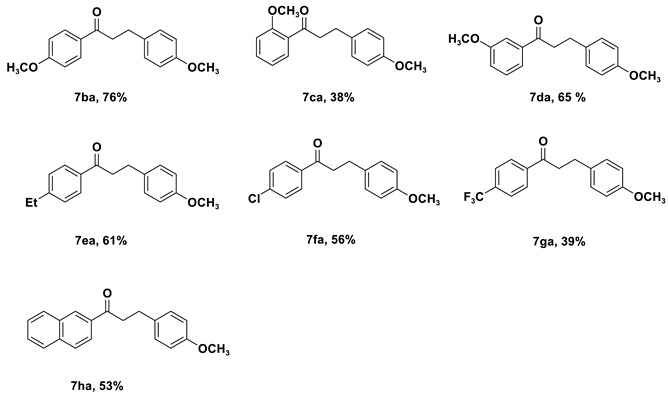

*^a^* All reactions were carried out using ketone (0.50 mmol), 4-methoxybenzyl alcohol (0.75 mmol), NaOH (50 mol%), plus catalyst **4c** (5.0 mol%) in toluene (2.0 mL) at 140 °C for 14 h. *^b^* Isolated yields.

**Table 5:** Crystal data and structure refinement parameters for complexes **4a** and **4c**.

	4a	4c·H_2_O
formula	C_20_H_15_MnN_4_O_4_	C_25_H_19_MnN_4_O_5_
*Mr*	430.30	492.4
temp (K)	297 (2)	296 (2)
crystsyst	Monoclinic	trigonal
space group	*P2(1)/c*	*R3c:H*
cryst size (mm)	0.24 × 0.22 × 0.08	0.20 × 0.18 × 0.08
*a* (Å)	14.4012 (8)	24.2887 (8)
*b* (Å)	10.2731 (5)	24.2887 (8)
*c* (Å)	13.1141 (6)	20.4273 (8)
*α* (deg)	90.00	90
*β* (deg)	101.735 (2)	90
*γ* (deg)	90	120
*V* (Å^3^)	1899.61 (17)	10,436.4 (8)
*Z*	4	18
*D*_calcd_ (g cm^−3^)	1.505	1.462
*μ* (mm^−1^)	0.073	0.614
*θ* range (deg)	3.21–27.57	2.75–27.51
index range	−18 ≤ h ≤ 18, −13 ≤ k ≤ 13, −17 ≤ l ≤ 17	−31 ≤ h ≤ 31, −31 ≤ k ≤ 31, −25 ≤ l ≤ 26
No. of data points collected	48,737	93,102
No. of unique data points	4337	5282
final R indices (*I* > 2*σ*(*I*)) R1	0.0736	0.0474
wR2	0.1829	0.1187
R indices (all data) R1	0.0795	0.0747
wR2	0.1852	0.1535
*F*(000)	880	4716.0
peak/hole (e·Å^−3^)	0.859/−0.572	0.871/−0.462
Goodness of fit on F^2^	1.390	1.083

## Data Availability

The original contributions presented in this study are included in the article/Supplementary Materials. Further inquiries can be directed to the corresponding author.

## Supplementary Materials

The following supporting information can be downloaded at: https://www.mdpi.com/article/10.3390/molecules30071465/s1.

## References

[1] Esfandiari M., Havaei G., Zahiri S., Mohammadnezhad G. (2022). Pincer complex immobilization onto different supports: Strategies and applications. Coord. Chem. Rev..

[2] Wang H., Wen J., Zhang X. (2021). Chiral tridentate ligands in transition metal-catalyzed asymmetric hydrogenation. Chem. Rev..

[3] Wu S., Wu Z., Ge Q., Zheng X., Yang Z. (2021). Antitumor activity of tridentate pincer and related metal complexes. Org. Biomol. Chem..

[4] Urgoitia G., Herrero M.T., Churruca F., Conde N., SanMartin R. (2021). Direct Arylation in the Presence of Palladium Pincer Complexes. Molecules.

[5] Liu J.-K., Gong J.-F., Song M.-P. (2019). Chiral palladium pincer complexes for asymmetric catalytic reactions. Org. Biomol. Chem..

[6] González-Sebastián L., Morales-Morales D. (2019). Cross-coupling reactions catalysed by palladium pincer complexes. A review of recent advances. J. Organomet. Chem..

[7] Selander N., Szabo K.J. (2011). Catalysis by palladium pincer complexes. Chem. Rev..

[8] Niu J.-L., Hao X.-Q., Gong J.-F., Song M.-P. (2011). Symmetrical and unsymmetrical pincer complexes with group 10 metals: Synthesis via aryl C–H activation and some catalytic applications. Dalton Trans..

[9] Keskin E., Arslan H. (2023). Palladium complexes containing NNN pincer type ligands and their activities in Suzuki-Miyaura cross coupling reaction. Heliyon.

[10] Sudharsan M., Nethaji M., Bhuvanesh N.S., Suresh D. (2021). Heteroleptic palladium(II) complexes of thiazolinyl-picolinamide derived NNN pincer ligand: An efficient catalyst for acylative suzuki coupling reactions. Asian J. Org. Chem..

[11] Lo P.-C., Yang C.-W., Wu W.-K., Chen C.-T. (2021). Synthesis, characterization, and catalytic application of palladium complexes containing indolyl-NNN-type ligands. Molecules.

[12] Shinde V.N., Bhuvanesh N., Kumar A., Joshi H. (2020). Design and syntheses of palladium complexes of NNN/CNN pincer ligands: Catalyst for cross dehydrogenative coupling reaction of heteroarenes. Organometallics.

[13] Kuyuldar S., Burda C., Connick W.B. (2019). Halide exchange studies of novel Pd(IIi) NNN-pincer complexes. RSC Adv..

[14] Guin A.K., Pal S., Chakraborty S., Chakraborty S., Paul N.D. (2023). N-alkylation of amines by C1–C10 aliphatic alcohols using a well-defined Ru(II)-catalyst. a metal–ligand cooperative approach. J. Org. Chem..

[15] Guin A.K., Mondal R., Chakraborty G., Pal S., Paul N.D. (2022). Ruthenium-catalyzed dehydrogenative functionalization of alcohols to pyrroles: A comparison between metal–ligand cooperative and non-cooperative approaches. J. Org. Chem..

[16] Nandi P.G., Jadi P.K., Das K., Prathapa S.J., Mandal B.B., Kumar A. (2021). Synthesis of NNN chiral ruthenium complexes and their cytotoxicity studies. Inorg. Chem..

[17] Bhattacharyya D., Sarmah B.K., Nandi S., Srivastava H.K., Das A. (2021). Selective catalytic synthesis of α-alkylated ketones and β-disubstituted ketones via acceptorless dehydrogenative cross-coupling of alcohols. Org. Lett..

[18] Maji M., Chakrabarti K., Paul B., Roy B.C., Kundu S. (2018). Ruthenium(II)-NNN-pincer-complex-catalyzed reactions between various alcohols and amines for sustainable C−N and C−C bond formation. Adv. Synth. Catal..

[19] Cao X.-N., Wan X.-M., Yang F.-L., Li K., Hao X.-Q., Shao T., Zhu X., Song M.-P. (2018). NNN pincer Ru(II)-complex-catalyzed α-alkylation of ketones with alcohols. J. Org. Chem..

[20] Teimouri M., Raju S., Acheampong E., Schmittou A.N., Donnadieu B., Wipf D.O., Pierce B.S., Stokes S.L., Emerson J.P. (2024). Aminoquinoline-based tridentate (NNN)-copper catalyst for C–N bond-forming reactions from aniline and diazo compounds. Molecules.

[21] Raju S., Teimouri M., Adhikari B., Donnadieu B., Stokes S.L., Emerson J.P. (2023). Copper complexes for the chemoselective N-arylation of arylamines and sulfanilamides via Chan–Evans–Lam cross-coupling. Dalton Trans..

[22] Zhou X., Yang J., Hao Z., Han Z., Lin J., Lu G.-L. (2023). Copper complexes with N,N,N-tridentate quinolinyl anilido-imine ligands: Synthesis and their catalytic application in Chan−Lam reactions. Molecules.

[23] Ataie S., Baker R.T. (2022). Comparing B-H bond activation in NiIIX(NNN)-catalyzed nitrile dihydroboration (X = Anionic N-, C-, O-, S-, or P-donor). Inorg. Chem..

[24] Subaramanian M., Midya S.P., Ramar P.M., Balaraman E. (2019). General synthesis of N-alkylation of amines with secondary alcohols via hydrogen autotransfer. Org. Lett..

[25] Patel U.N., Pandey D.K., Gonnade R.G., Punji B. (2016). Synthesis of quinoline-based NNN-pincer nickel(II) complexes: A robust and improved catalyst system for C–H bond alkylation of azoles with alkyl halides. Organometallics.

[26] Cheng Z., Li M., Zhang X.-Y., Sun Y., Yu Q.-L., Zhang X.-H., Lu Z. (2023). Cobalt-catalyzed regiodivergent double hydrosilylation of arylacetylenes. Angew. Chem. Int. Ed..

[27] Pattanaik S., Kumar A., Gunanathan C. (2023). Cobalt-catalysed [1,2]-Wittig rearrangement of ethers to secondary alcohols. Chem. Commun..

[28] Chen J., Ying J., Lu Z. (2022). Cobalt-catalyzed branched selective hydroallylation of terminal alkynes. Nat. Commun..

[29] Banach Ł., Brykczynska D., Gorczynski A., Wyrzykiewicz B., Skrodzki M., Pawluc P. (2022). Markovnikov-selective double hydrosilylation of challenging terminal aryl alkynes under cobalt and iron catalysis. Chem. Commun..

[30] Pradhan D.R., Pattanaik S., Kishore J., Gunanathan C. (2020). Cobalt-catalyzed acceptorless dehydrogenation of alcohols to carboxylate salts and hydrogen. Org. Lett..

[31] Pal S., Das S., Chakraborty S., Khanra S., Paul N.D. (2023). Zn(II)-catalyzed multicomponent sustainable synthesis of pyridines in air. J. Org. Chem..

[32] Das S., Mondal R., Chakraborty G., Guin A.K., Das A., Paul N.D. (2021). Zinc stabilized azo-anion radical in dehydrogenative synthesis of N-heterocycles. an exclusively ligand centered redox controlled approach. ACS Catal..

[33] Mukherjee A., Nerush A., Leitus G., Shimon L.J.W., Ben David Y., Espinosa Jalapa N.A., Milstein D. (2016). Manganese-catalyzed environmentally benign dehydrogenative coupling of alcohols and amines to form aldimines and H_2_: A catalytic and mechanistic study. J. Am. Chem. Soc..

[34] Sklyaruk J., Borghs J.C., El-Sepelgy O., Rueping M. (2019). Catalytic C_1_ alkylation with methanol and isotope-labeled methanol. Angew. Chem. Int. Ed..

[35] Fu S., Shao Z., Wang Y., Liu Q. (2017). Manganese-catalyzed upgrading of ethanol into 1-butanol. J. Am. Chem. Soc..

[36] Espinosa-Jalapa N.A., Kumar A., Leitus G., Diskin-Posner Y., Milstein D. (2017). Synthesis of cyclic imides by acceptorless dehydrogenative coupling of diols and amines catalyzed by a manganese pincer complex. J. Am. Chem. Soc..

[37] Widegren M.B., Harkness G.J., Slawin A.M.Z., Cordes D.B., Clarke M.L. (2017). A highly active manganese catalyst for enantioselective ketone and ester hydrogenation. Angew. Chem. Int. Ed..

[38] Kallmeier F., Irrgang T., Dietel T., Kempe R. (2016). Highly active and selective manganese C=O bond hydrogenation catalysts: The importance of the multidentate ligand, the ancillary ligands, and the oxidation state. Angew. Chem. Int. Ed..

[39] Das U.K., Janes T., Kumara A., Milstein D. (2020). Manganese catalyzed selective hydrogenation of cyclic imides to diols and amines. Green Chem..

[40] Zhang L., Tang Y., Han Z., Ding K. (2019). Lutidine-based chiral pincer manganese catalysts for enantioselective hydrogenation of ketones. Angew. Chem. Int. Ed..

[41] Homberg L., Roller A., Hultzsch K.C. (2019). A highly active PN^3^ manganese pincer complex performing N-alkylation of amines under mild conditions. Org. Lett..

[42] Ling F., Hou H., Chen J., Nian S., Yi X., Wang Z., Song D., Zhong W. (2019). Highly enantioselective synthesis of chiral benzhydrols via manganese catalyzed asymmetric hydrogenation of unsymmetrical benzophenones using an imidazole-based chiral PNN tridentate ligand. Org. Lett..

[43] Wang Y., Zhu L., Shao Z., Li G., Lan Y., Liu Q. (2019). Unmasking the ligand effect in manganese-catalyzed hydrogenation: Mechanistic insight and catalytic application. J. Am. Chem. Soc..

[44] Brzozowska A., Zubar V., Ganardi R.-C., Rueping M. (2020). Chemoselective hydroboration of propargylic alcohols and amines using a manganese(II) catalyst. Org. Lett..

[45] Vasilenko V., Blasius C.K., Wadepohl H., Gade L.H. (2017). Mechanism-based enantiodivergence in manganese reduction catalysis: A chiral pincer complex for the highly enantioselective hydroboration of ketones. Angew. Chem. Int. Ed..

[46] Ma X., Zuo Z., Liu G., Huang Z. (2017). Manganese-catalyzed asymmetric hydrosilylation of aryl ketones. ACS Omega.

[47] Zhang G., Zeng H., Wu J., Yin Z., Zheng S., Fettinger J.C. (2016). Highly selective hydroboration of alkenes, ketones and aldehydes catalyzed by a well-defined manganese complex. Angew. Chem. Int. Ed..

[48] Xu Y., Wang T., Li Y., Li S., Zhang Y., Wang J., Li F., Jiang H. (2024). NNN pincer palladium(II) complexes with N-(2-(1H-pyrazol-1-yl)phenyl)-picolinamide ligands: Synthesis, characterization, and application to heck coupling reaction. Transition Met Chem..

[49] Wang T., Hao X.-Q., Huang J.-J., Wang K., Gong J.-F., Song M.-P. (2014). Chiral CNN pincer palladium(II) complexes with 2-aryl-6-(oxazolinyl)pyridine ligands: Synthesis, characterization, and application to enantioselective allylation of isatins and Suzuki–Miyaura coupling reaction. Organometallics.

[50] Wang T., Hao X.-Q., Zhang X.-X., Gong J.-F., Song M.-P. (2011). Synthesis, structure and catalytic properties of CNN pincer palladium(ii) and ruthenium(IIi) complexes with N-substituted-2-aminomethyl-6-phenylpyridines. Dalton Trans..

[51] Wang T., Guo J., Wang X., Guo H., Jia D., Wang H., Liu L. (2019). Cross coupling of benzylammonium salts with boronic acids using a well-defined N-heterocyclic carbene-palladium(II) precatalyst. RSC Adv..

[52] Wang T., Xu K., Meng T., Zhang A., Wang H., Shen S., Liu L. (2017). N-Heterocyclic carbene-palladium(II) complexes with acridine ligand: Synthesis, characterization and catalytic applications. Chin. J. Org. Chem..

[53] Wang T., Liu L., Xu K., Xie H., Shen H., Zhao W.-X. (2016). Synthesis and characterization of trinuclear N-heterocyclic carbene-palladium(II) complexes and their applications in the Suzuki-Miyaura cross-coupling reaction. RSC Adv..

[54] Wang T., Xie H., Liu L., Zhao W.-X. (2016). N-heterocyclic carbene-palladium(II) complexes with benzoxazole or benzothiazole ligands: Synthesis, characterization, and application to Suzuki-Miyaura aross-coupling reaction. J. Organomet. Chem..

[55] Wang T., Wang Y., Xu K., Zhang Y., Guo J., Liu L. (2021). Transition-metal-free DMAP-mediated aromatic esterification of amides with organoboronic acids. Eur. J. Org. Chem..

[56] Wang T., Wang X., Song Y., Huo J., Zhou J., Kang Q., Liu L. (2021). Cu(OAc)_2_-Mediated C-H bond dithiolation of amide-oxazolines with aryl thiols. Chin. J. Org. Chem..

[57] Daw P., Kumar A., Espinosa-Jalapa N.A., Diskin-Posner Y., Ben-David Y., Milstein D. (2018). Synthesis of pyrazines and quinoxalines via acceptorless dehydrogenative coupling routes catalyzed by manganese pincer complexes. ACS Catal..

[58] Singh A., Maji A., Joshi M., Choudhury A.R., Ghosh K. (2021). Designed pincer ligand supported Co(II)-based catalysts for dehydrogenative activation of alcohols: Studies on N-alkylation of amines, α-alkylation of ketones and synthesis of quinolines. Dalton Trans..

[59] Singh S., Saini R., Chaudhary V.K., Ghosh K. (2025). Organometallic Ru(III) catalysts for α-alkylation of carbonyl compounds using alcohols: Mechanistic insights via detection of key intermediates. Chem. Asian J..

[60] Deshmukh G., Murugavel R. (2024). Bimetallic Ru(II) complex catalysed β-alkylation of secondary alcohols and α-alkylation of ketones: Selective formation of saturated ketones. Eur. J. Org. Chem..

[61] Samanta A., Behera P., Chaubey A., Mondal A., Pal D., Mohar K., Roy L., Srimani D. (2024). Experimental and theoretical insights for designing Zn^2+^ complexes to trigger chemo-selective hetero-coupling of alcohols. Chem. Commun..

[62] Xu Q., Chen J., Tian H., Yuan X., Li S., Zhou C., Liu J. (2014). Catalyst-free dehydrative α-alkylation of ketones with alcohols: Green and selective autocatalyzed synthesis of alcohols and ketones. Angew. Chem. Int. Ed..

[63] Ouyang K., Xi Z. (2013). Roles of bases in transition-metal catalyzed organic reactions. Acta Chim. Sinica..

[64] Sheldrick G.M. (1997). SHELXS-97, Program for Crystal Structure Solution.

[65] Sheldrick G.M. (1997). SHELXL-97, Program for Crystal Structure Refinement.

