# Supplementary material for: Synthesis, Characterization and Application of NNN Pincer Manganese Complexes with Pyrazole Framework in α-Alkylation Reaction
Source: Molecules. 2025 Mar 26;30(7):1465. doi: 10.3390/molecules30071465 (PMC11990691; doi:10.3390/molecules30071465)
Supplement: Supplementary file 1 [file molecules-30-01465-s001.zip › molecules-3506156-supplementary.pdf]

Supporting Information

Synthesis, characterization and application of NNN pincer manganese complexes with pyrazole framework in α-alkylation reaction

Tao Wang[1*], Yongli Xu[2], Mengxin Du[2], Zhiyuan Hu[1], Lantao Liu[1*]

[1]Henan Engineering Laboratory of Green Synthesis for Pharmaceuticals, School of Chemistry and Chemical Engineering, Shangqiu Normal University, Shangqiu, Henan, 476000, People's Republic of China

[2]College of Petrochemical Engineering, Liaoning Petrochemical University, Fushun 113001, People's Republic of China

Copies of the ^{1}H and ^{13}C NMR spectra of new complexes **4** ……….................................S-2

Characterization data of the catalysis products **7**………...S-5

Copies of the ^{1}H and ^{13}C NMR spectra of the catalysis products **7** ……….......................S-8

*Tel./Fax: +86 0370-2595126; e-mail: wt67751726@126.com or liult05@iccas.ac.cn .

Copies of the ^{1}H and ^{13}C NMR spectra of new complexes 4

^{1}H NMR spectra of complex **4a**

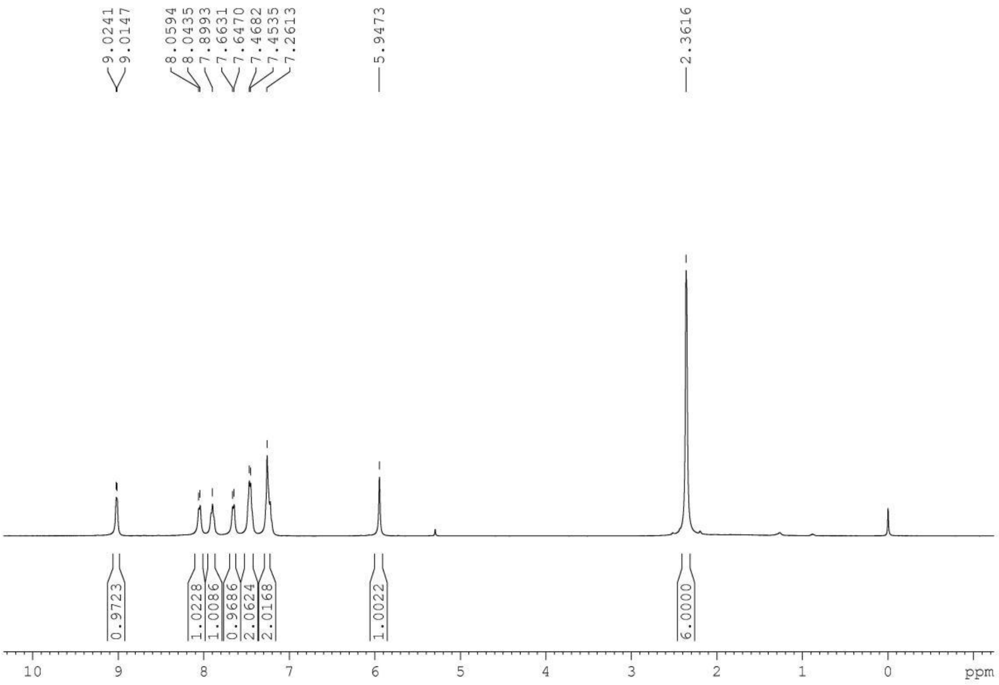

^{13}C NMR spectra of complex **4a**

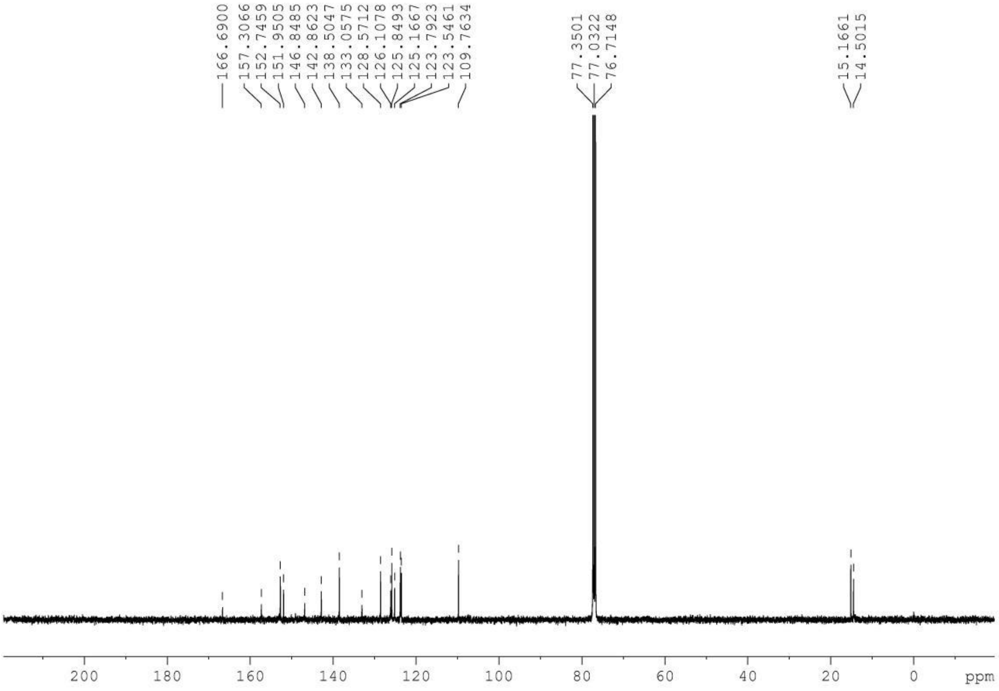

^{1}H NMR spectra of complex **4b**

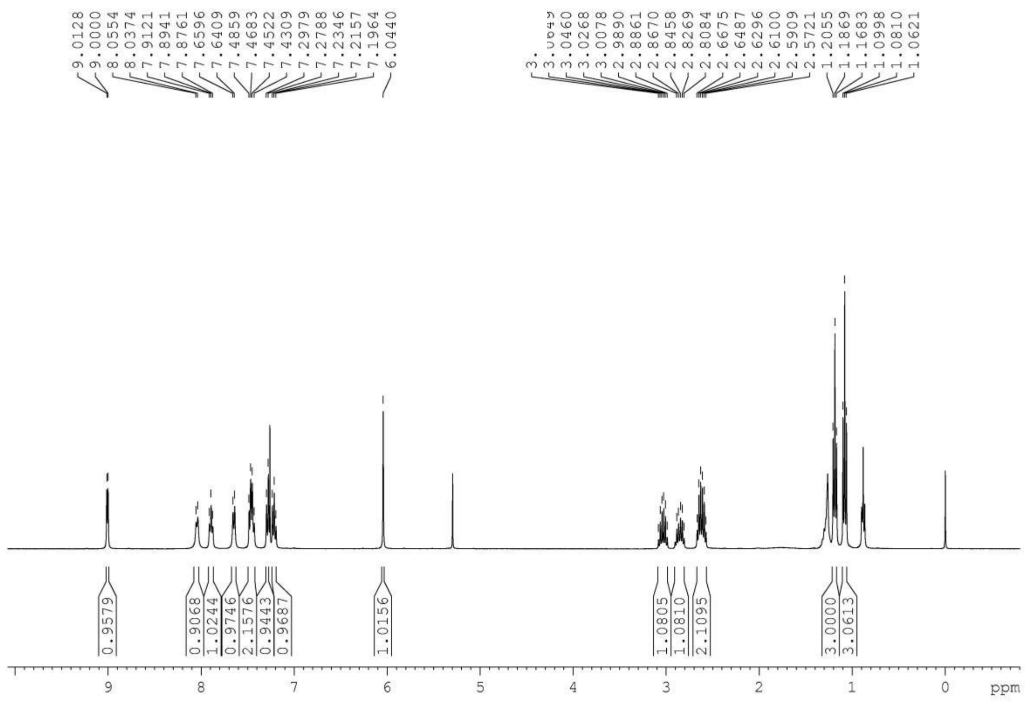

^{13}C NMR spectra of complex **4b**

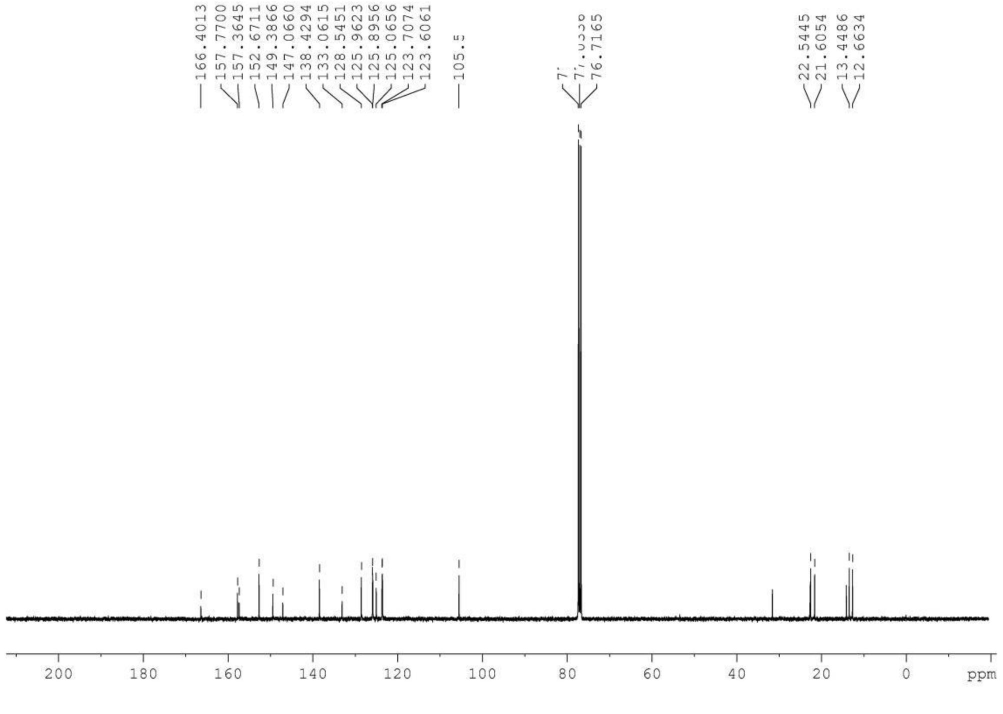

^{1}H NMR spectra of complex **4c**

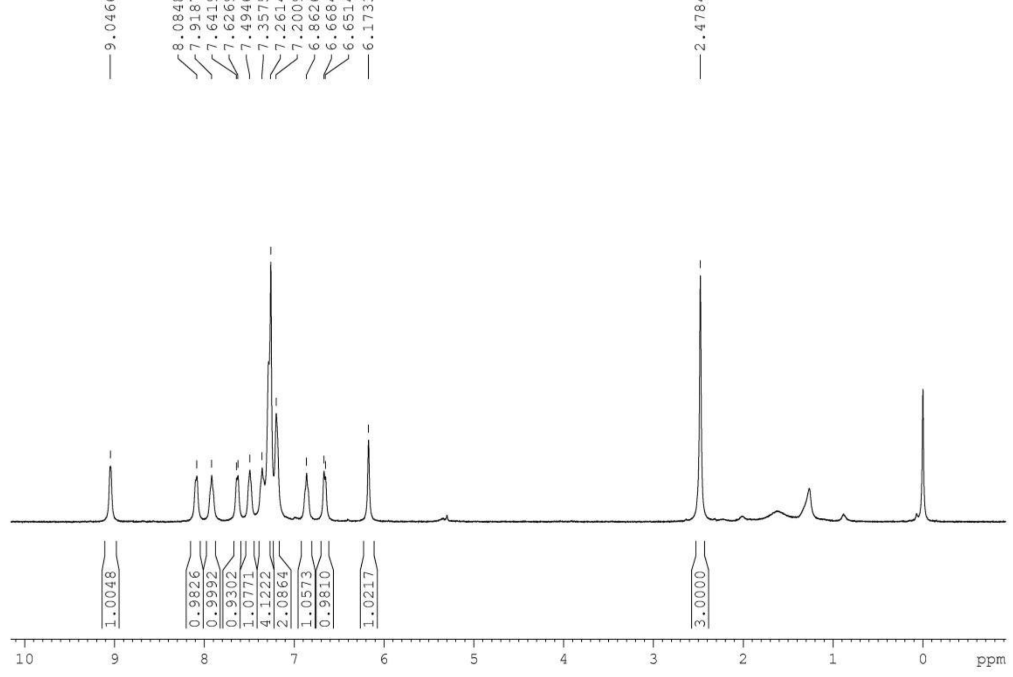

^{13}C NMR spectra of complex **4c**

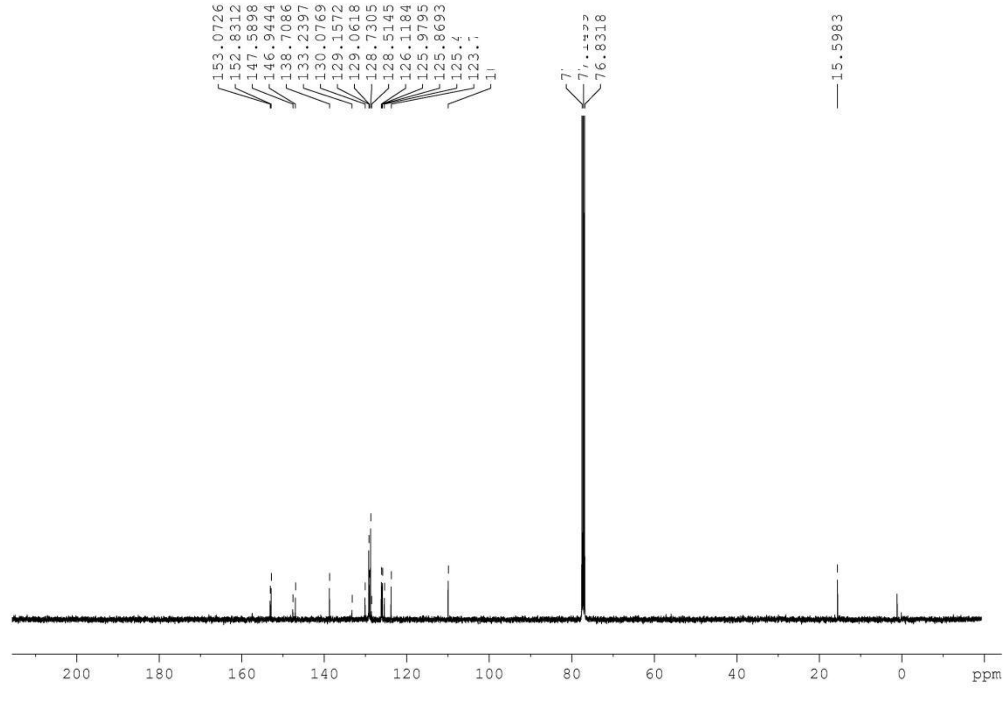

Characterization data of the catalysis products 7

3-(4-methoxyphenyl)-1-phenylpropan-1-one (7aa): 97.2 mg, 81% yield. ^{1}H NMR (400 MHz, $CDCl_3$) δ 7.96-7.94 (m, 2H), 7.57-7.53 (m, 1H), 7.45 (t, *J* = 8.1 Hz, 2H), 7.17 (d, *J* = 8.6 Hz, 2H), 6.84 (d, *J* = 8.6 Hz, 2H), 3.79 (s, 3H), 3.27 (t, *J* = 7.3 Hz, 2H), 3.01 (t, *J* = 8.0 Hz, 2H). ^{13}C NMR (100 MHz, $CDCl_3$) δ 199.4, 158.0, 136.9, 133.3, 133.1, 129.4, 128.6, 128.1, 113.9, 55.3, 40.7, 29.3.

3-(2-methoxyphenyl)-1-phenylpropan-1-one (7ab): 66.4 mg, 56% yield. ^{1}H NMR (400 MHz, $CDCl_3$) δ 7.96 (d, *J* = 7.2 Hz, 2H), 7.45 (t, *J* = 7.4 Hz, 1H), 7.42 (t, *J* = 7.7 Hz, 2H), 7.21-7.17 (m, 2H), 6.90-6.83 (m, 2H), 3.80 (s, 3H), 3.25 (t, *J* = 7.2 Hz, 2H), 3.04 (t, *J* = 8.1 Hz, 2H). ^{13}C NMR (100 MHz, $CDCl_3$) δ 200.0, 157.6, 137.0, 132.9, 130.2, 129.5, 128.6, 128.1, 127.6, 120.6, 110.3, 55.2, 39.0, 25.7.

3-(3-methoxyphenyl)-1-phenylpropan-1-one (7ac): 69.9 mg, 56% yield. ^{1}H NMR (400 MHz, $CDCl_3$) δ 7.96 (d, *J* = 7.2 Hz, 2H), 7.56 (t, *J* = 7.4 Hz, 1H), 7.45 (t, *J* = 7.8 Hz, 2H), 7.22 (d, *J* = 7.2 Hz, 1H), 6.85 (d, *J* = 7.6 Hz, 1H), 6.80 (s, 1H), 6.76 (dd, *J* = 8.2, 2.1 Hz, 1H), 3.80 (s, 3H), 3.31 (t, *J* = 7.3 Hz, 2H), 3.05 (t, *J* = 8.0 Hz, 2H). ^{13}C NMR (100 MHz, $CDCl_3$) δ 199.2, 159.7, 142.9, 136.8, 133.1, 129.5, 128.6, 128.1, 120.8, 114.2, 111.4, 55.2, 40.4, 30.2.

1-phenyl-3-(p-tolyl)propan-1-one (7ad): 89.3 mg, 80% yield. ^{1}H NMR (400 MHz, $CDCl_3$) δ 7.96-7.94 (m, 2H), 7.57-7.53 (m, 1H), 7.45 (t, *J* = 7.8 Hz, 2H), 7.13 (q, *J* = 8.1, 16.2 Hz, 4H), 3.28 (t, *J* = 7.3 Hz, 2H), 3.03 (t, *J* = 8.0 Hz, 2H), 2.32 (s, 3H). ^{13}C NMR (100 MHz, $CDCl_3$) δ 199.4, 138.2, 136.9, 135.6, 133.0, 129.2, 128.6, 128.3, 128.1, 40.6, 29.7, 21.0.

3-(4-(tert-butyl)phenyl)-1-phenylpropan-1-one (7ae): 98.6 mg, 74% yield. ^{1}H NMR (400 MHz, $CDCl_3$) δ 7.96 (d, *J* = 7.2 Hz, 2H), 7.57-7.52 (m, 1H), 7.44 (t, *J* = 7.8 Hz, 2H), 7.32 (d, *J* = 8.2 Hz, 2H), 7.19 (d, *J* = 8.2 Hz, 2H), 3.29 (t, *J* = 7.3 Hz, 2H), 3.04 (t, *J* = 8.0 Hz, 2H), 1.31 (s, 9H). ^{13}C NMR (100 MHz, $CDCl_3$) δ 199.4, 149.0, 138.2, 136.9, 133.0, 128.6, 128.1, 128.0, 125.4, 40.5, 34.4, 31.4, 29.6.

3-(4-chlorophenyl)-1-phenylpropan-1-one (7af): 85.0 mg, 70% yield. ^{1}H NMR (400 MHz, $CDCl_3$) δ 7.95 (d, *J* = 7.2 Hz, 2H), 7.57 (t, *J* = 7.4 Hz, 1H), 7.46 (t, *J* = 7.7 Hz, 2H), 7.25 (d, *J* = 4.2 Hz, 2H), 7.18 (d, *J* = 8.4 Hz, 2H), 3.29 (t, *J* = 7.3 Hz, 2H), 3.04 (t, *J* = 7.6 Hz, 2H).

^{13}C NMR (100 MHz, $CDCl_3$) δ 198.9, 139.7, 136.7, 133.2, 131.9, 129.8, 128.7, 128.6, 128.0, 40.2, 29.4.

3-(naphthalen-1-yl)-1-phenylpropan-1-one (7ag): 117.2 mg, 90% yield. ^1H NMR (400 MHz, $CDCl_3$) δ 8.02 (d, J = 8.2 Hz, 1H), 7.91-7.89 (m, 2H), 7.83 (d, J = 7.6 Hz, 1H), 7.71-7.68 (m, 1H), 7.50-7.44 (m, 3H), 7.39-7.36 (m, 4H), 3.50 (t, J = 7.1 Hz, 2H), 3.36 (t, J = 8.5 Hz, 2H). ^{13}C NMR (100 MHz, $CDCl_3$) δ 199.3, 137.4, 136.8, 134.0, 133.2, 131.8, 129.0, 128.7, 128.1, 127.1, 126.2, 126.2, 125.7, 125.7, 123.6, 39.8, 27.2.

3-(naphthalen-2-yl)-1-phenylpropan-1-one (7ah): 103.5 mg, 79% yield. ^1H NMR (400 MHz, $CDCl_3$) δ 7.95 (d, J = 7.3 Hz, 2H), 7.77 (d, J = 7.4 Hz, 2H), 7.67 (s, 1H), 7.52 (d, J = 7.4 Hz, 2H), 7.44-7.7.35 (m, 5H), 3.35 (t, J = 7.1 Hz, 2H), 3.21 (t, J = 7.8 Hz, 2H). ^{13}C NMR (100 MHz, $CDCl_3$) δ 199.2, 159.7, 142.9, 136.8, 133.1, 129.5, 128.6, 128.0, 120.8, 114.2, 111.4, 40.4, 30.1.

1,3-bis(4-methoxyphenyl)propan-1-one (7ba): 102.7 mg, 76% yield. ^1H NMR (400 MHz, $CDCl_3$) δ 7.92 (d, J = 8.9 Hz, 2H), 7.15 (d, J = 8.6 Hz, 2H), 6.90 (d, J = 8.6 Hz, 2H), 6.82 (d, J = 8.6 Hz, 2H), 3.83 (s, 3H), 3.76 (s, 3H), 3.19 (t, J = 7.3 Hz, 2H), 2.98 (t, J = 8.0 Hz, 2H). ^{13}C NMR (100 MHz, $CDCl_3$) δ 198.0, 163.4, 158.0, 133.5, 130.3, 130.0, 129.4, 113.9, 113.7, 55.5, 55.2, 40.4, 29.5.

1-(2-methoxyphenyl)-3-(4-methoxyphenyl)propan-1-one (7ca): 51.6 mg, 38% yield. ^1H NMR (400 MHz, $CDCl_3$) δ 7.67 (dd, J = 7.6, 1.5 Hz, 1H), 7.45-7.41 (m, 1H), 7.14 (d, J = 8.5 Hz, 2H), 6.99 (d, J = 7.9 Hz, 1H), 6.94 (d, J = 8.4 Hz, 1H), 6.82 (d, J = 8.5 Hz, 2H), 3.86 (s, 3H), 3.77 (s, 3H), 3.26 (t, J = 7.4 Hz, 2H), 2.95 (t, J = 8.0 Hz, 2H). ^{13}C NMR (100 MHz, $CDCl_3$) δ 201.9, 158.5, 157.8, 133.8, 133.4, 130.4, 129.4, 120.7, 114.3, 113.8, 111.5, 55.5, 55.3, 45.7, 29.6.

1-(3-methoxyphenyl)-3-(4-methoxyphenyl)propan-1-one (7da): 87.9 mg, 65% yield. ^1H NMR (400 MHz, $CDCl_3$) δ 7.51 (d, J = 7.6 Hz, 1H), 7.47 (s, 1H), 7.33 (t, J = 8.0 Hz, 1H), 7.15 (d, J = 8.6 Hz, 2H), 7.08 (dd, J = 8.2, 1.8 Hz, 1H), 6.83 (d, J = 8.6 Hz, 2H), 3.82 (s, 3H), 3.76 (s, 3H), 3.23 (t, J = 7.3 Hz, 2H), 2.99 (t, J = 7.8 Hz, 2H). ^{13}C NMR (100 MHz, $CDCl_3$) δ 199.2, 159.9, 158.0, 138.3, 133.3, 129.6, 129.4, 120.7, 119.6, 114.0, 112.3, 55.5, 55.3, 40.8, 29.4.

1-(4-ethylphenyl)-3-(4-methoxyphenyl)propan-1-one (7ea): 82.0 mg, 61% yield. ^1H

NMR (400 MHz, $CDCl_3$) δ 7.87 (d, J = 8.3 Hz, 2H), 7.25 (d, J = 8.3 Hz, 2H), 7.16 (d, J = 8.6 Hz, 2H), 6.83 (d, J = 8.6 Hz, 2H), 3.77 (s, 3H), 3.23 (t, J = 7.3 Hz, 2H), 2.99 (t, J = 8.0 Hz, 2H), 2.68 (q, J = 15.2, 7.6, Hz, 2H), 1.24 (t, J = 7.6 Hz, 3H). ^{13}C NMR (100 MHz, $CDCl_3$) δ 199.1, 158.0, 150.0, 134.7, 133.5, 129.4, 128.3, 128.1, 113.9, 55.3, 40.6, 29.4, 28.9, 15.2.

1-(4-chlorophenyl)-3-(4-methoxyphenyl)propan-1-one (7fa): 76.4 mg, 56% yield. ^{1}H NMR (400 MHz, $CDCl_3$) δ 7.86 (d, J = 8.6 Hz, 2H), 7.39 (d, J = 8.6 Hz, 2H), 7.14 (d, J = 8.6, 2H), 6.82 (d, J = 8.6 Hz, 2H), 3.77 (s, 3H), 3.21 (t, J = 7.3 Hz, 2H), 2.99 (t, J = 7.8 Hz, 2H). ^{13}C NMR (100 MHz, $CDCl_3$) δ 198.1, 158.1, 139.5, 135.2, 133.1, 129.5, 129.4, 128.9, 114.0, 55.3, 40.7, 29.2.

3-(4-methoxyphenyl)-1-(4-(trifluoromethyl)phenyl)propan-1-one (7ga): 59.4 mg, 39% yield. ^{1}H NMR (400 MHz, $CDCl_3$) δ 8.03 (d, J = 8.1 Hz, 2H), 7.70 (d, J = 8.2 Hz, 2H), 7.16 (d, J = 8.6, 2H), 6.84 (d, J = 8.6 Hz, 2H), 3.78 (s, 3H), 3.28 (t, J = 7.3 Hz, 2H), 3.02 (t, J = 7.7 Hz, 2H). ^{13}C NMR (100 MHz, $CDCl_3$) δ 198.4, 158.1, 139.5, 134.3 (q, J = 32.6 Hz), 132.9, 129.4, 128.4, 125.7 (q, J = 3.7 Hz), 123.6 (q, J = 271.1 Hz), 114.0, 55.3, 41.0, 29.1.

3-(4-methoxyphenyl)-1-(naphthalen-2-yl)propan-1-one (7ha): 76.9 mg, 53% yield. ^{1}H NMR (400 MHz, $CDCl_3$) δ 8.41 (s, 1H), 8.0 (dd, J = 8.6, 1.7 Hz, 1H), 7.89 (d, J = 8.0 Hz, 1H), 7,85-7,81 (m, 2H), 7.57-7.48 (m, 2H), 7.18 (d, J = 8.5 Hz, 2H), 6.84 (d, J = 8.6 Hz, 2H), 3.76 (s, 3H), 3.36 (t, J = 7.3 Hz, 2H), 3.05 (t, J = 7.9 Hz, 2H). ^{13}C NMR (100 MHz, $CDCl_3$) δ 199.3, 158.0, 135.6, 134.2, 133.4, 132.6, 132.0, 129.7, 129.6, 129.4, 128.5, 127.8, 126.8, 123.9, 114.0, 55.3, 40.8, 29.5.

Copies of the ^{1}H and ^{13}C NMR spectra of the catalysis products 7

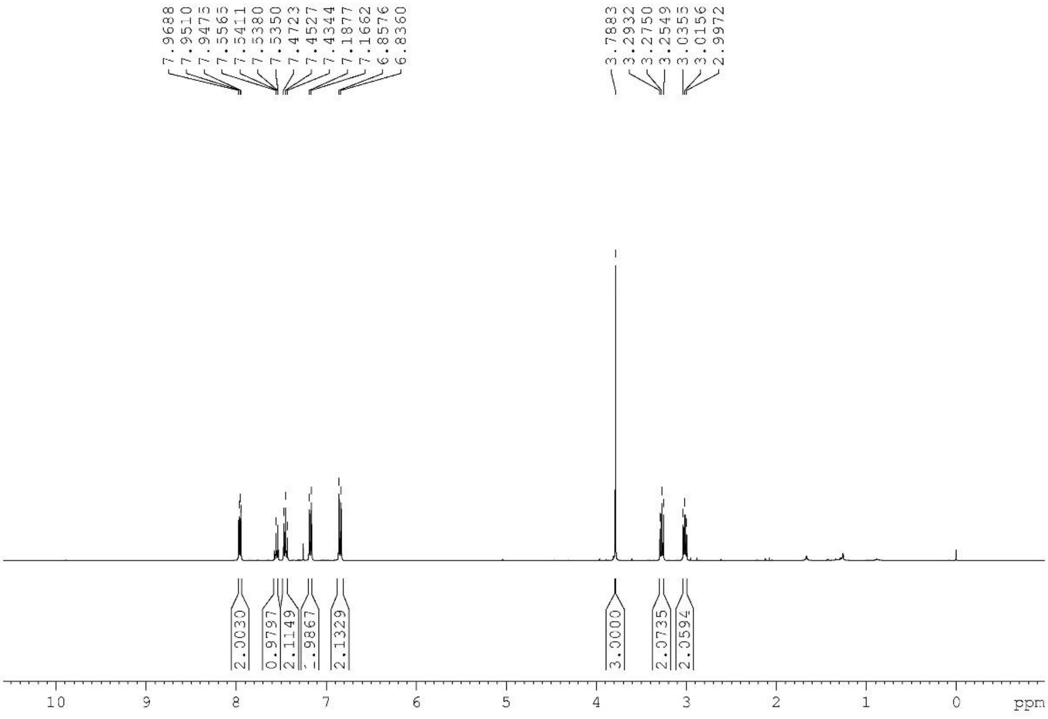

^{1}H NMR spectrum of compound 7aa ($CDCl_3$, 400M)

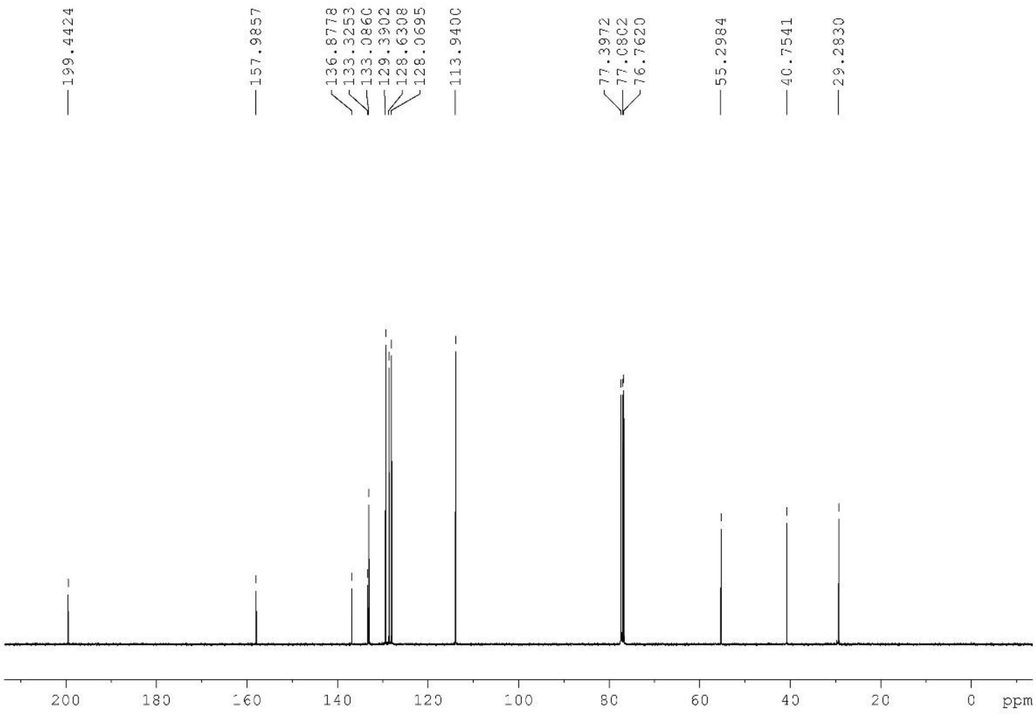

^{13}C NMR spectrum of compound 7aa ($CDCl_3$, 100M)

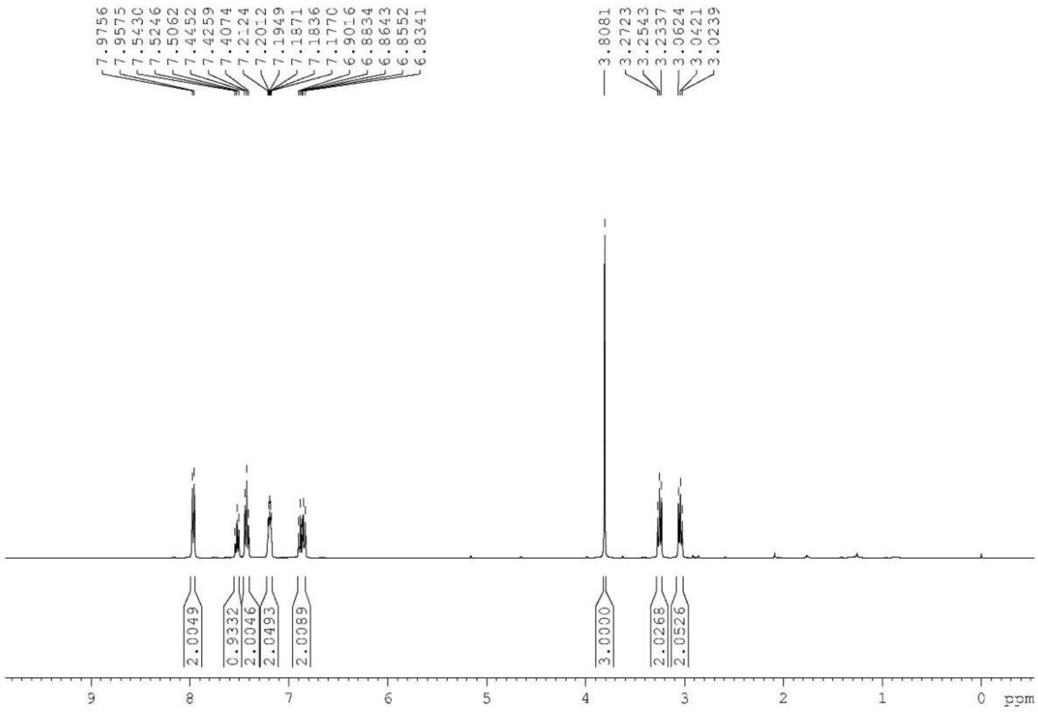

^{1}H NMR spectrum of compound 7ab (CDCl$_3$, 400M)

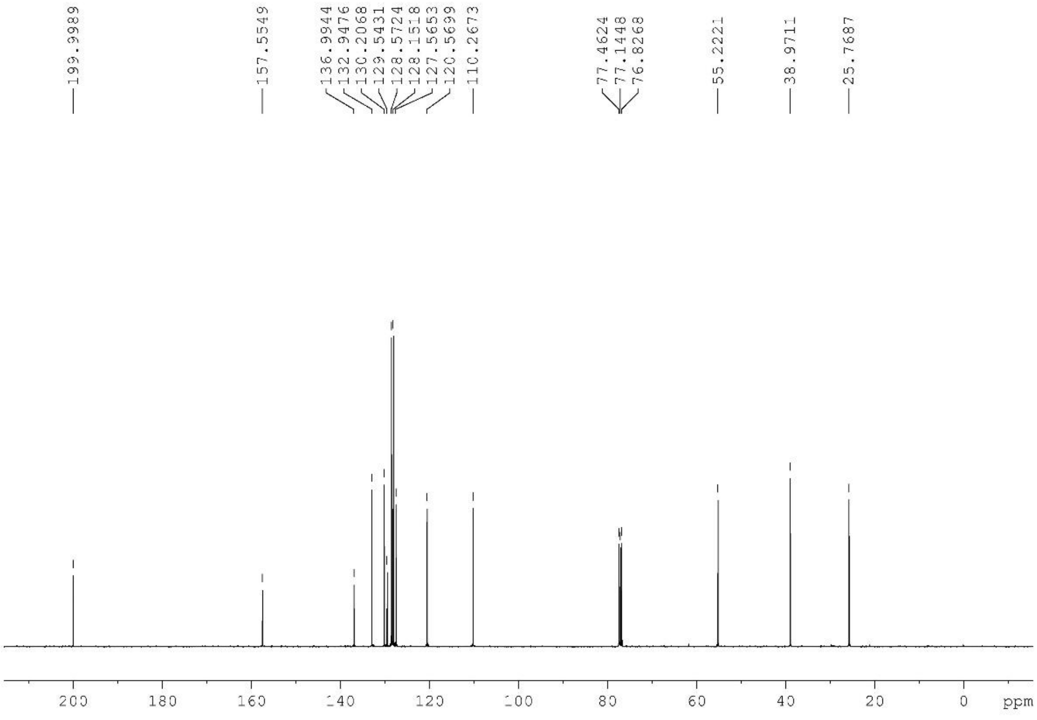

^{13}C NMR spectrum of compound 7ab (CDCl$_3$, 100M)

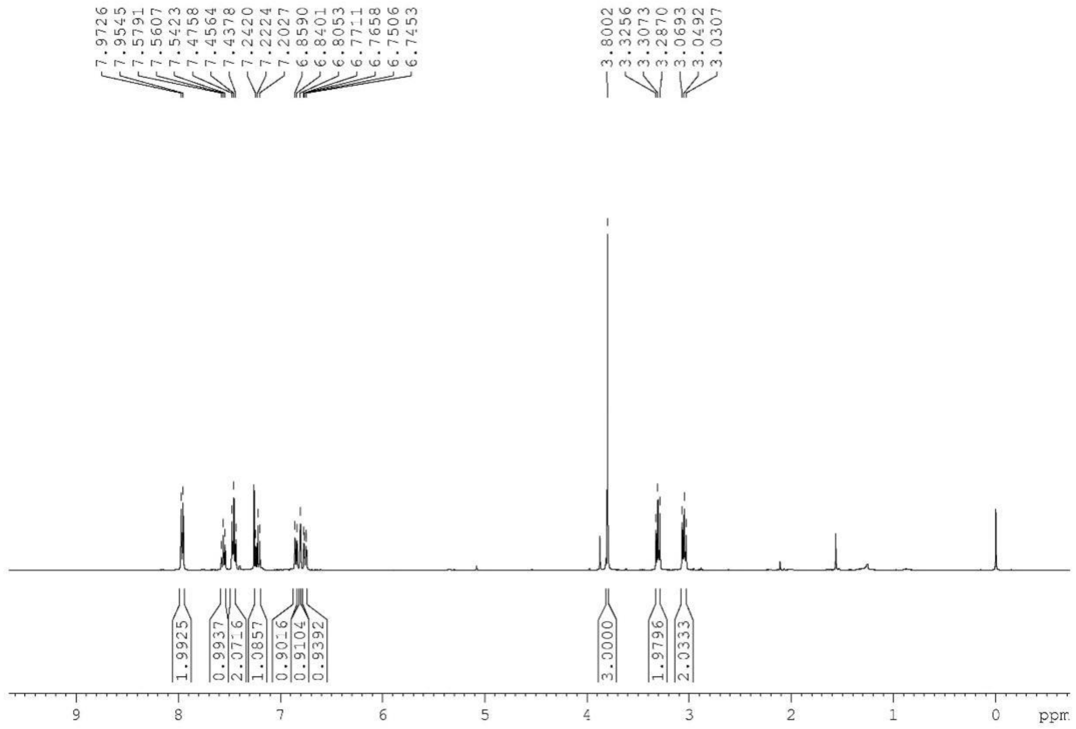

^{1}H NMR spectrum of compound 7ac (CDCl$_3$, 400M)

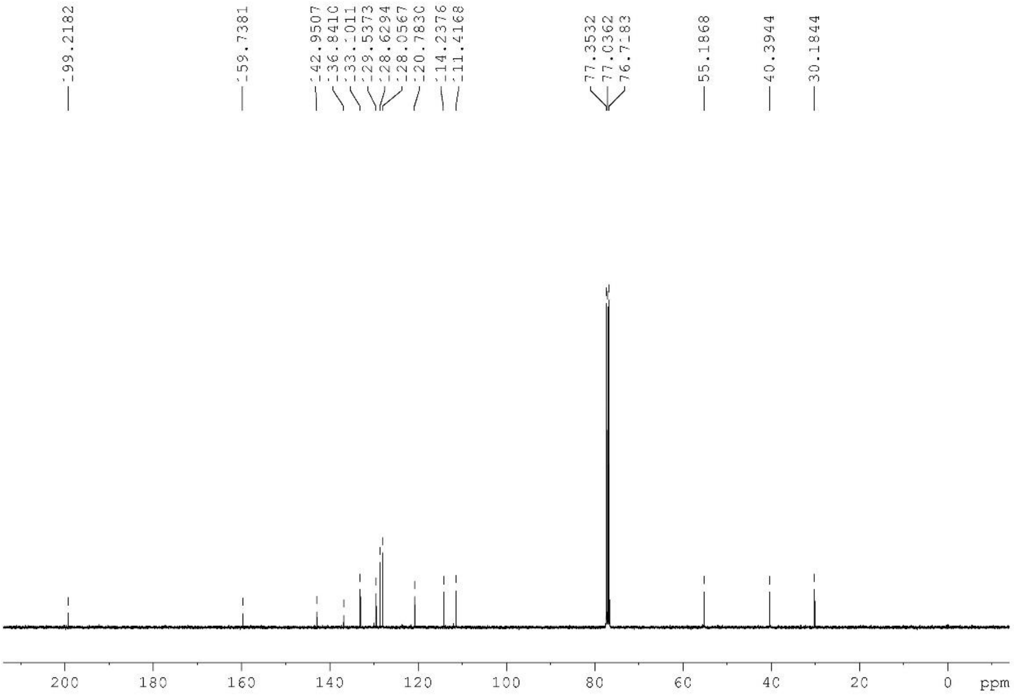

^{13}C NMR spectrum of compound 7ac (CDCl$_3$, 100M)

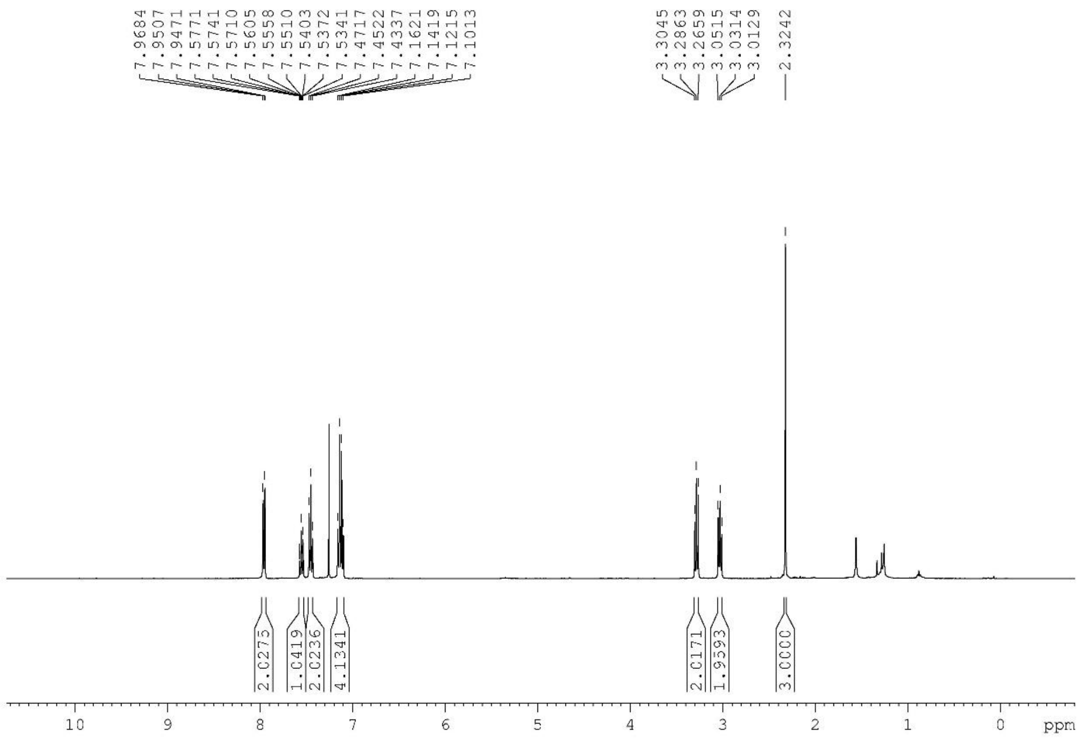

^{1}H NMR spectrum of compound 7ad ($CDCl_3$, 400M)

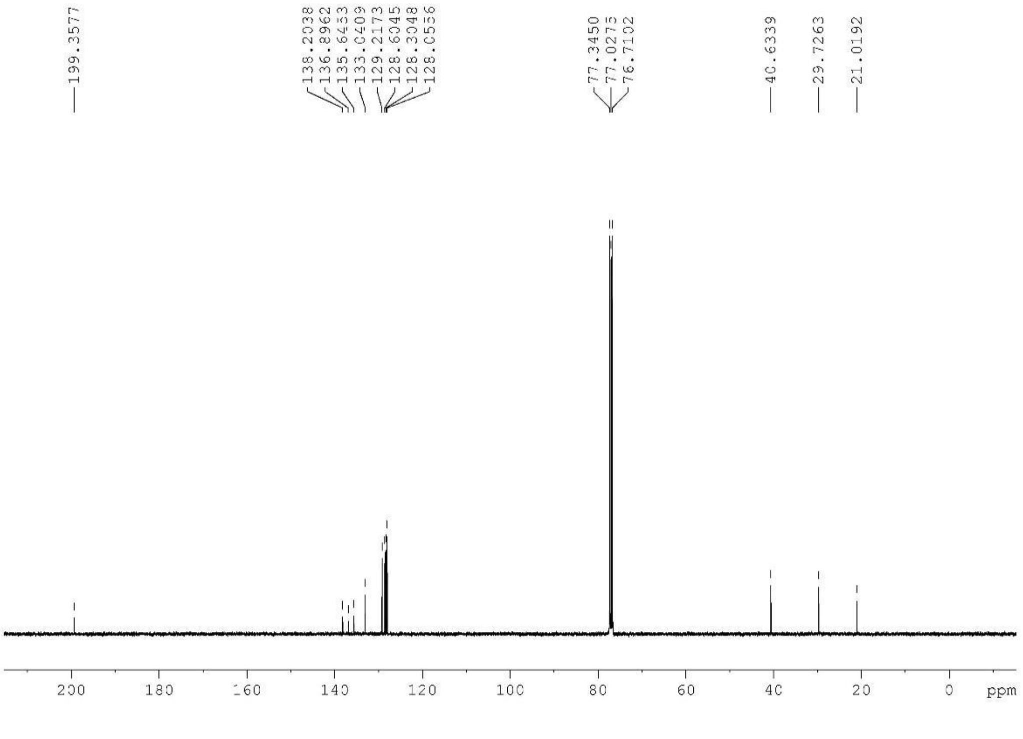

^{13}C NMR spectrum of compound 7ad ($CDCl_3$, 100M)

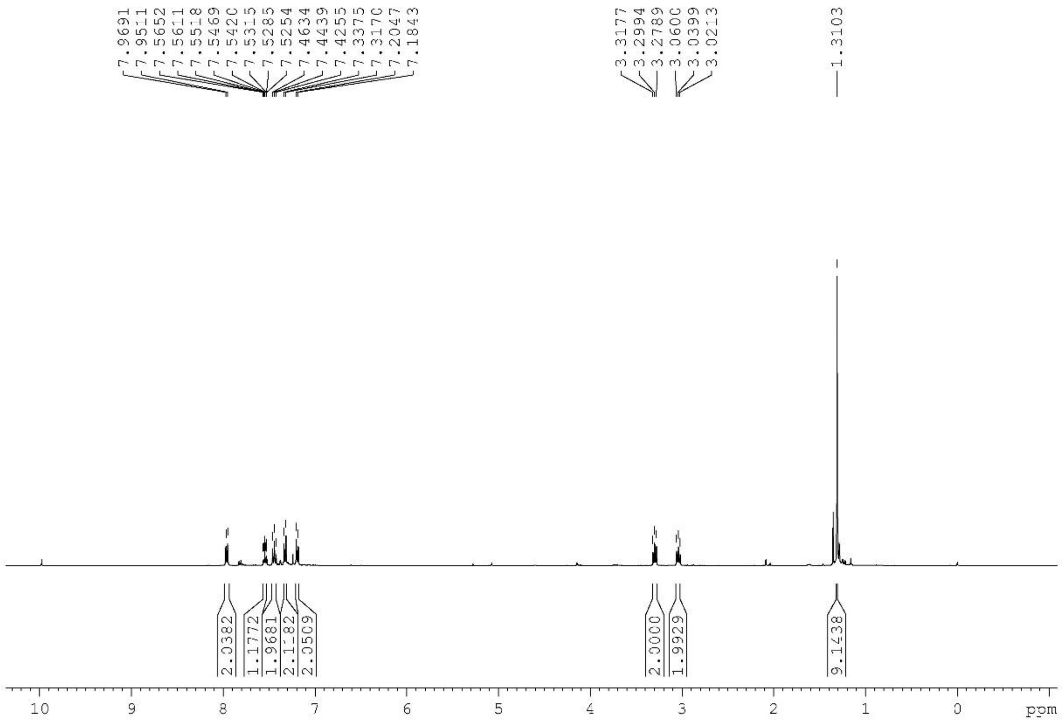

^{1}H NMR spectrum of compound 7ae ($CDCl_3$, 400M)

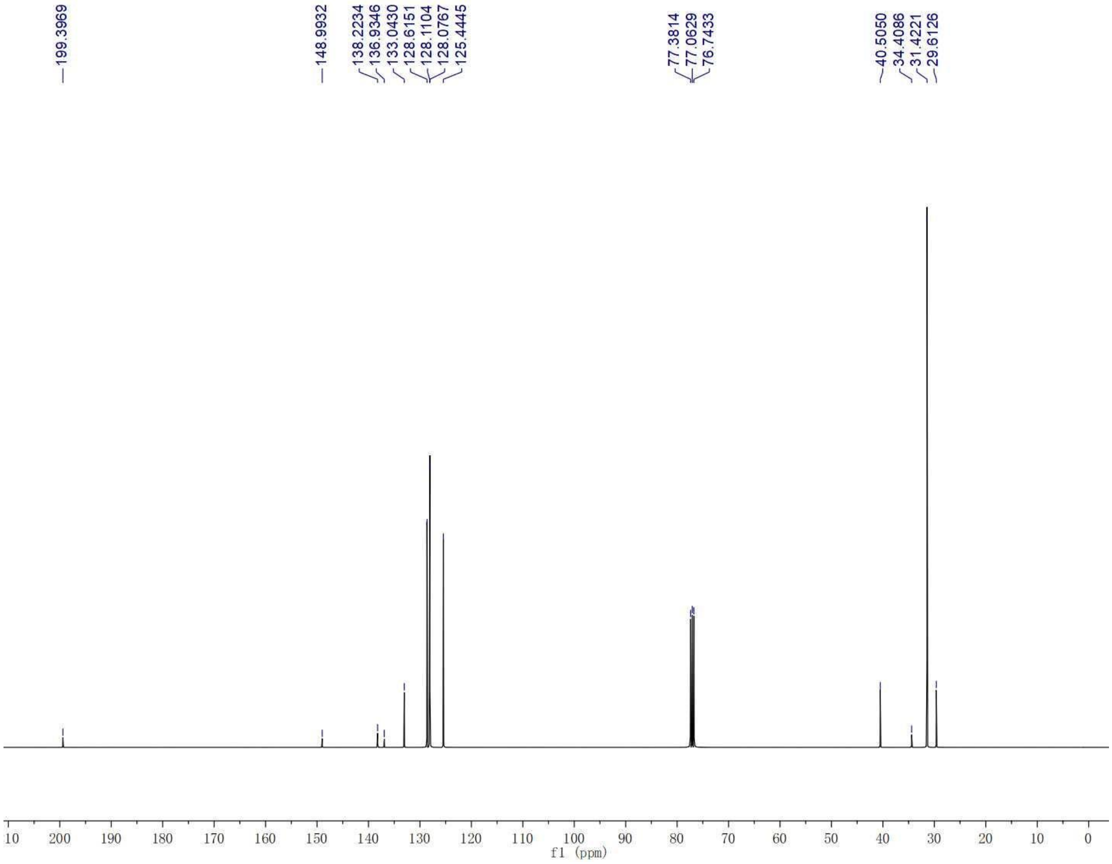

^{13}C NMR spectrum of compound 7ae ($CDCl_3$, 100M)

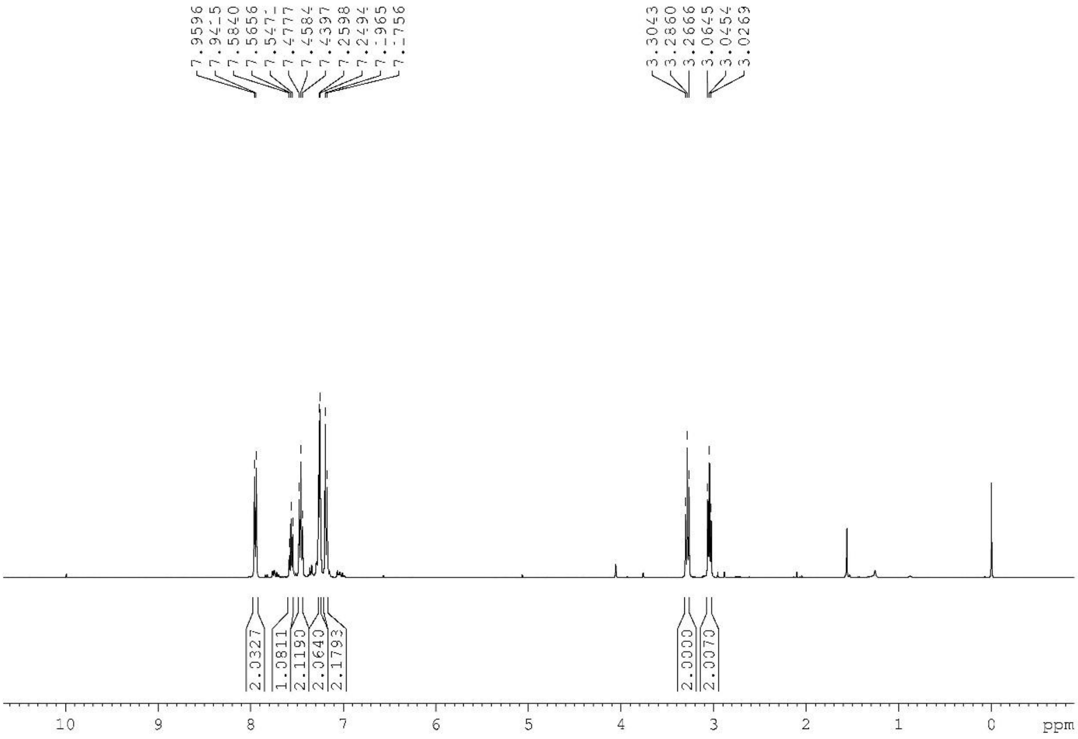

^{1}H NMR spectrum of compound 7af ($CDCl_3$, 400M)

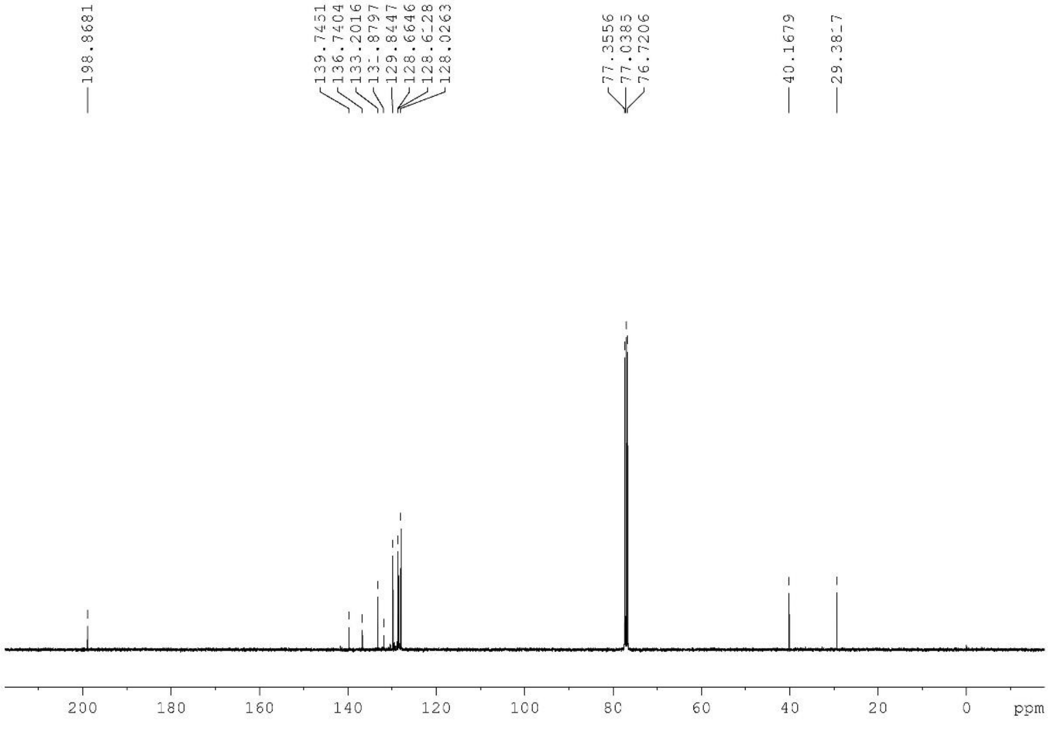

^{13}C NMR spectrum of compound 7af (CDCl$_3$, 100M)

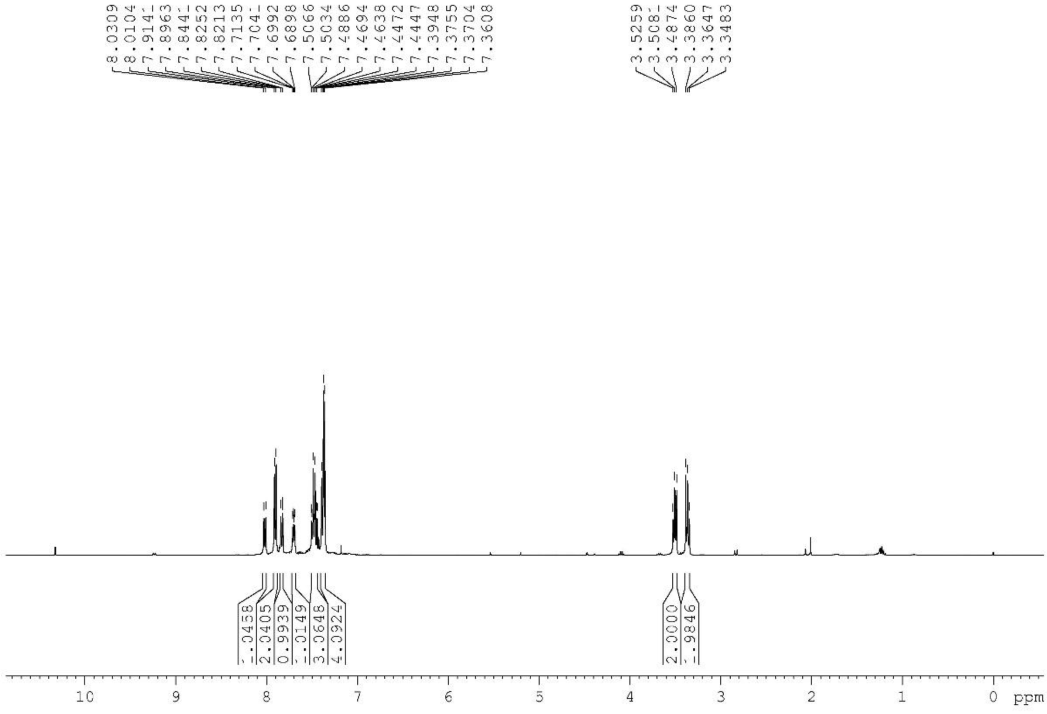

^{1}H NMR spectrum of compound 7ag (CDCl$_3$, 400M)

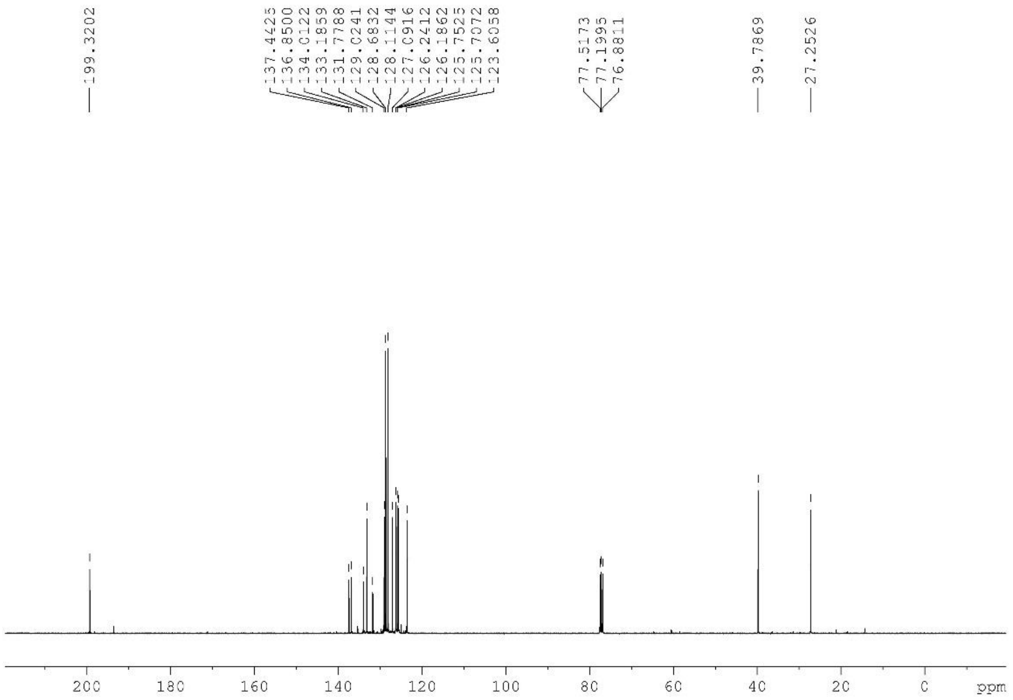

^{13}C NMR spectrum of compound 7ag (CDCl$_3$, 100M)

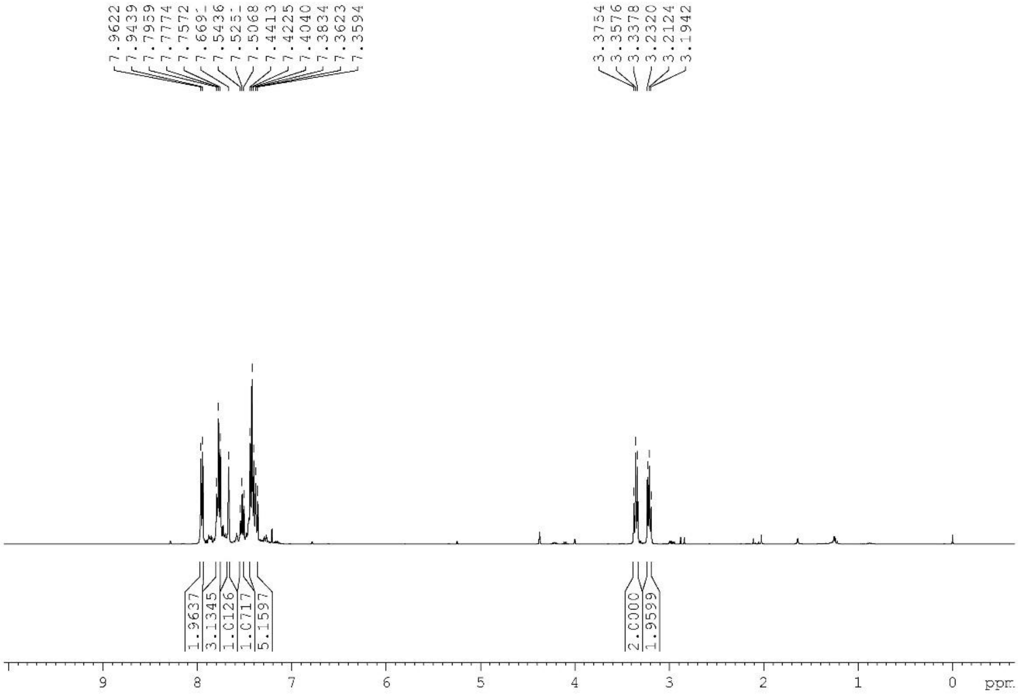

^{1}H NMR spectrum of compound 7ah (CDCl$_3$, 400M)

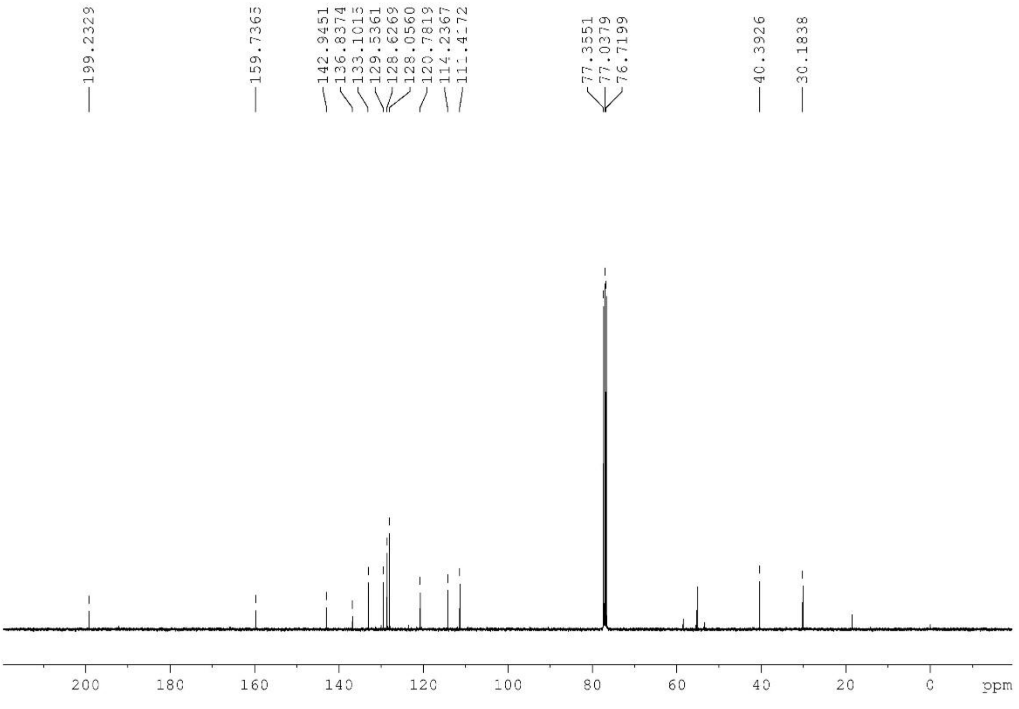

^{13}C NMR spectrum of compound 7ah (CDCl$_3$, 100M)

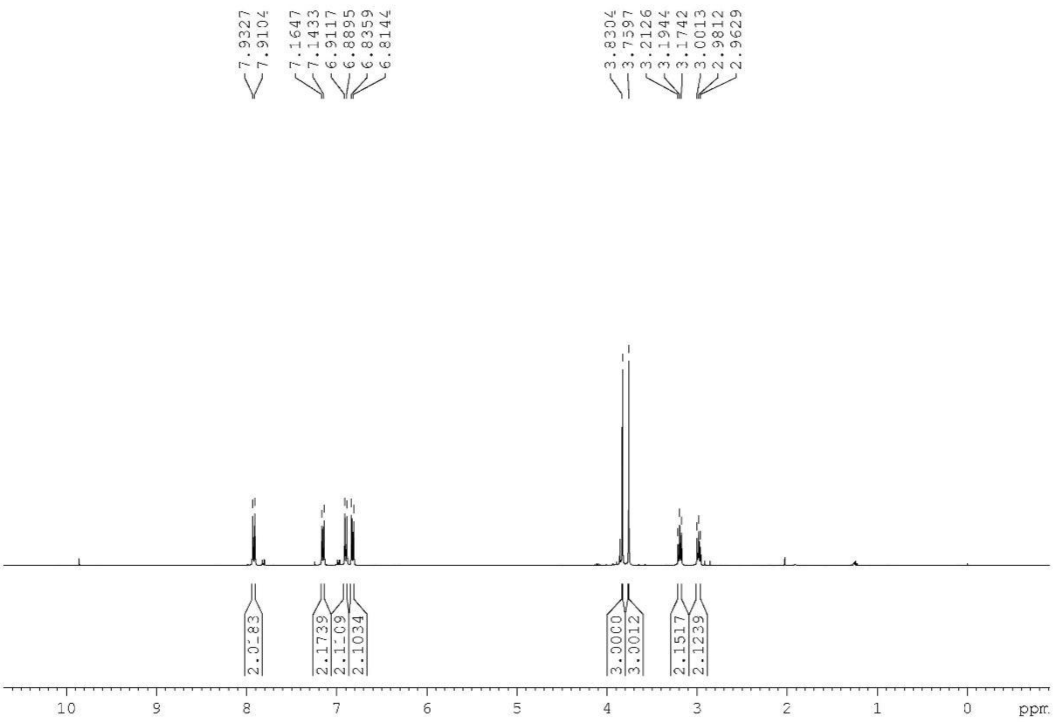

^{1}H NMR spectrum of compound 7ba (CDCl$_3$, 400M)

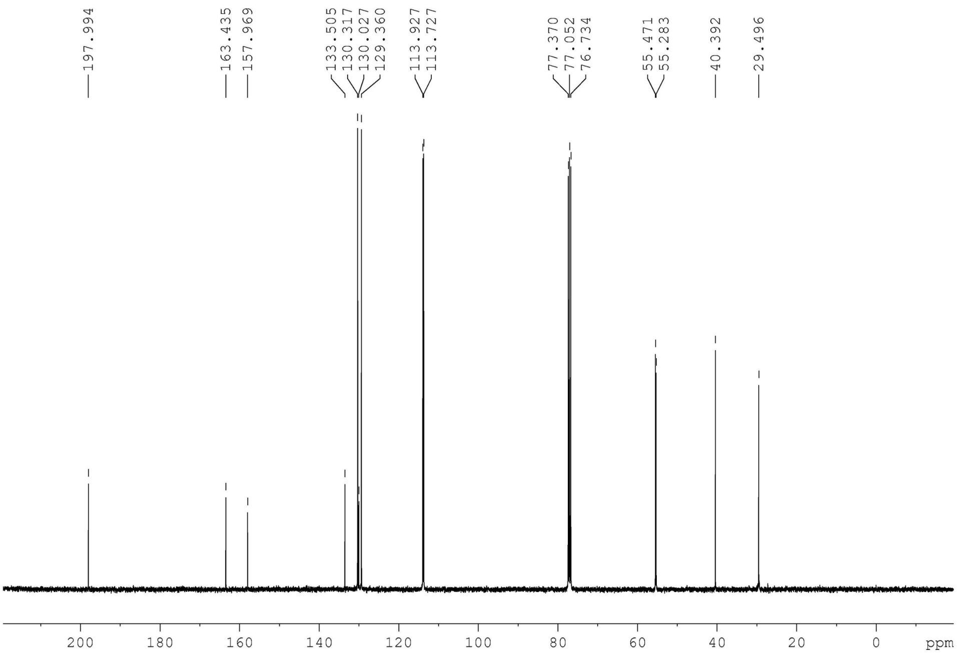

^{13}C NMR spectrum of compound 7ba (CDCl$_3$, 100M)

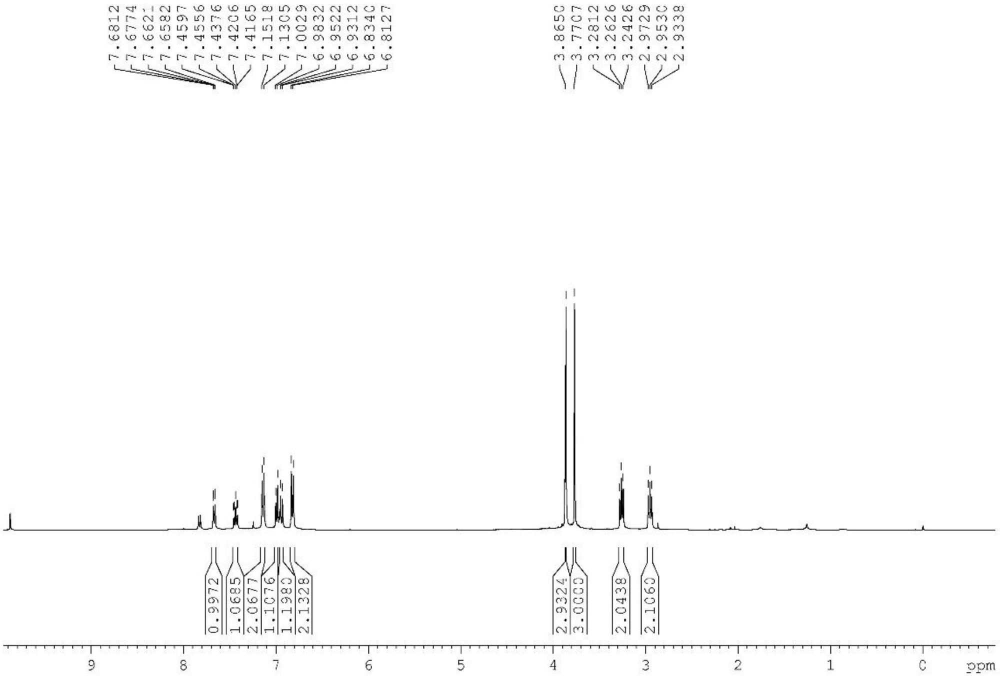

^{1}H NMR spectrum of compound 7ca ($CDCl_3$, 400M)

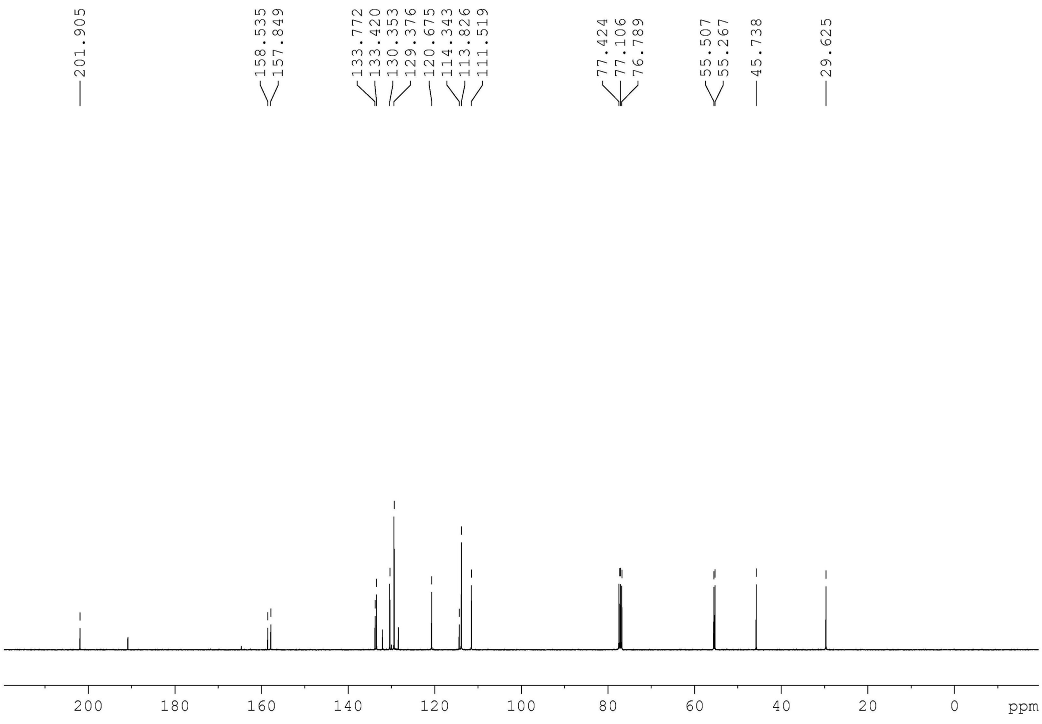

^{13}C NMR spectrum of compound 7ca ($CDCl_3$, 100M)

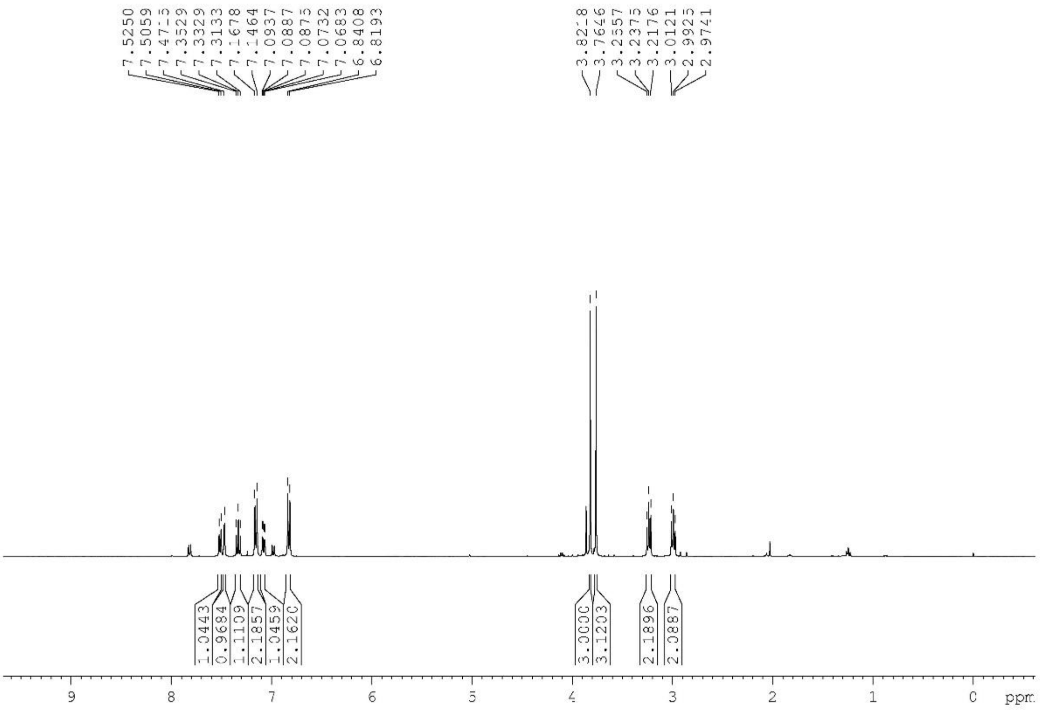

^{1}H NMR spectrum of compound 7da (CDCl$_3$, 400M)

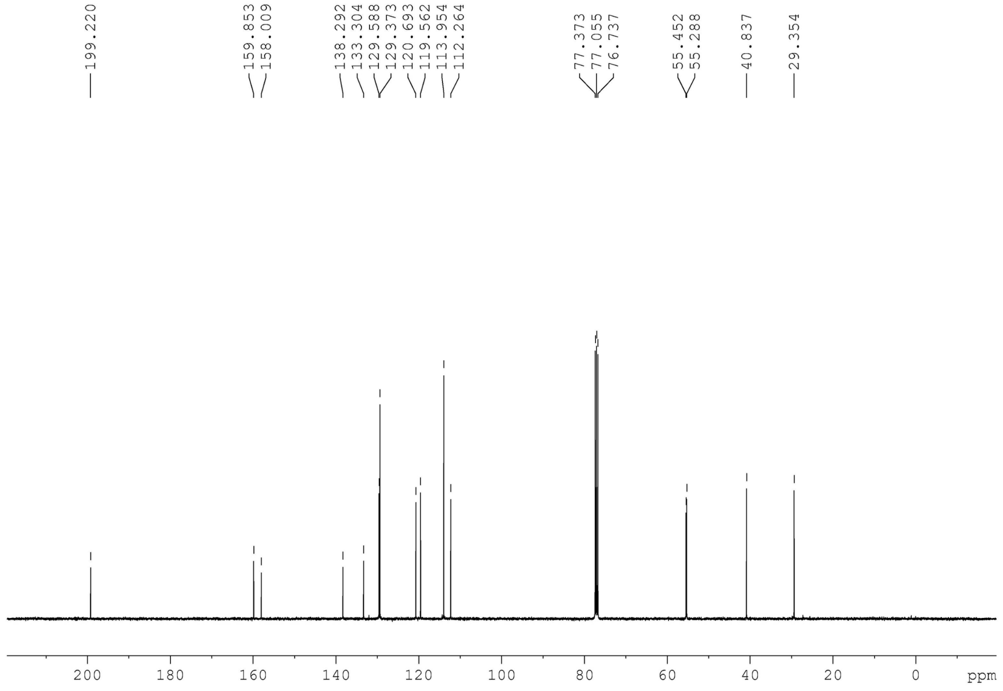

^{13}C NMR spectrum of compound 7da (CDCl$_3$, 100M)

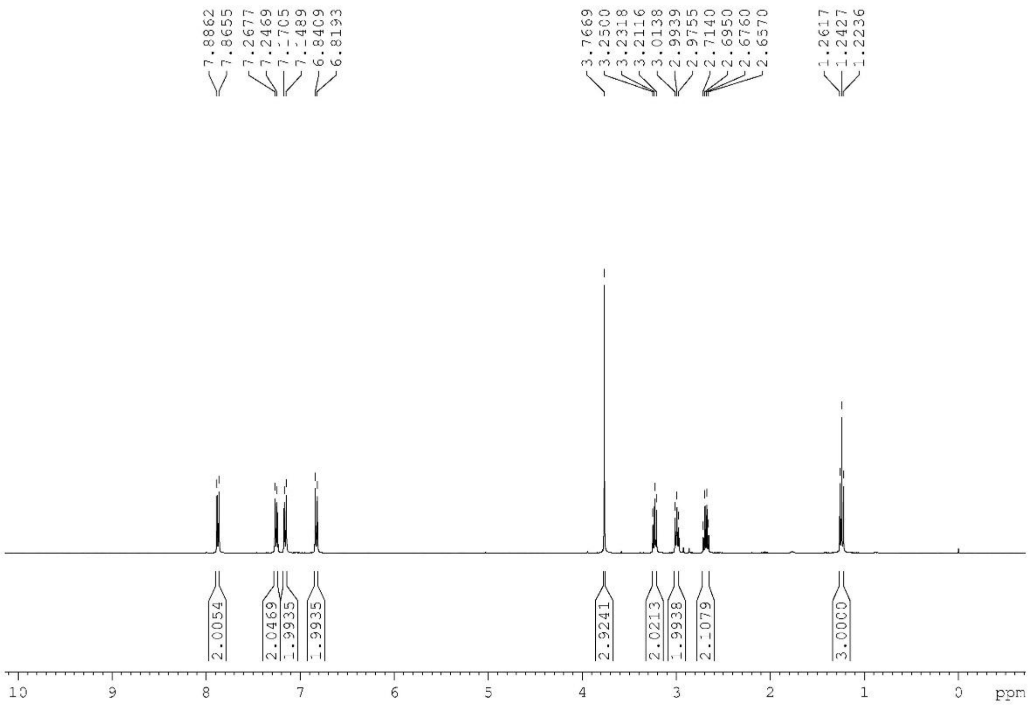

^{1}H NMR spectrum of compound 7ea ($CDCl_3$, 400M)

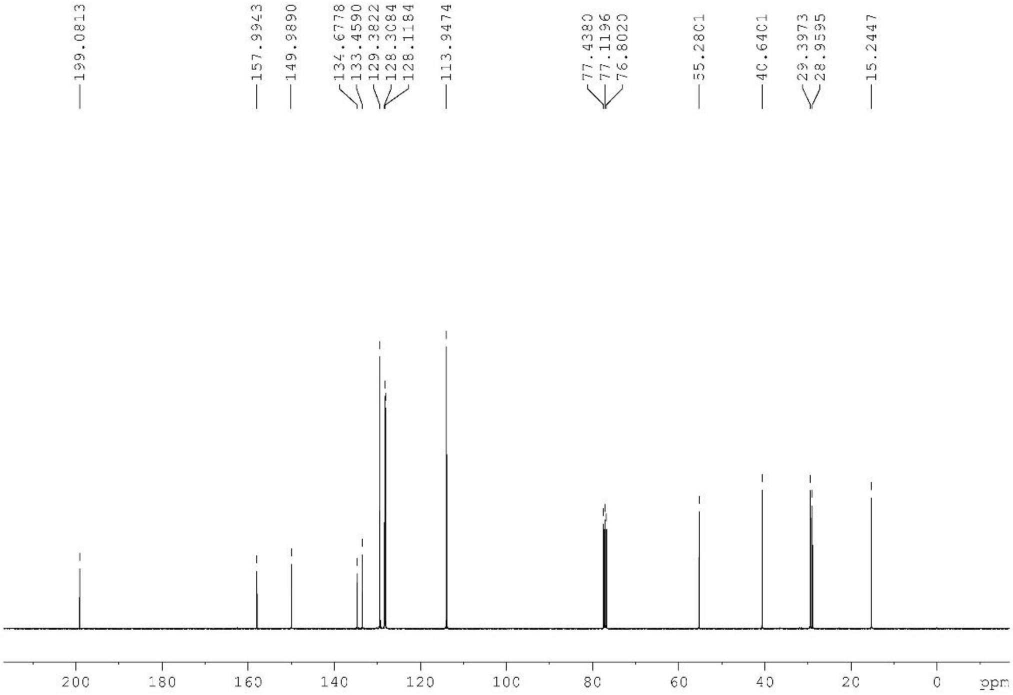

^{13}C NMR spectrum of compound 7ea ($CDCl_3$, 100M)

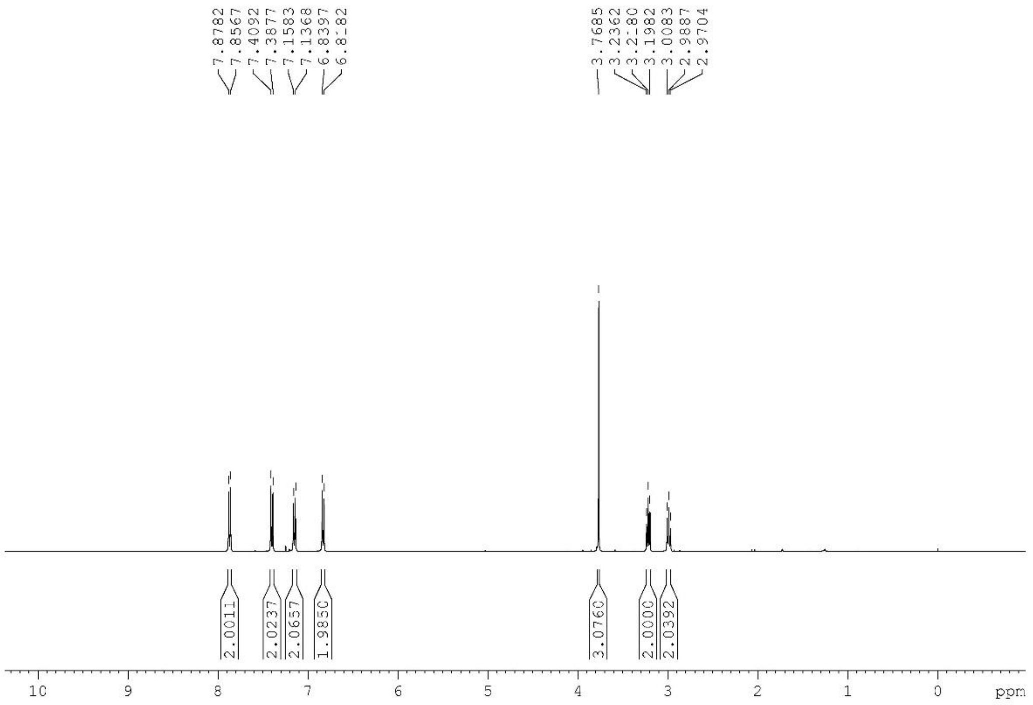

^{1}H NMR spectrum of compound 7fa ($CDCl_3$, 400M)

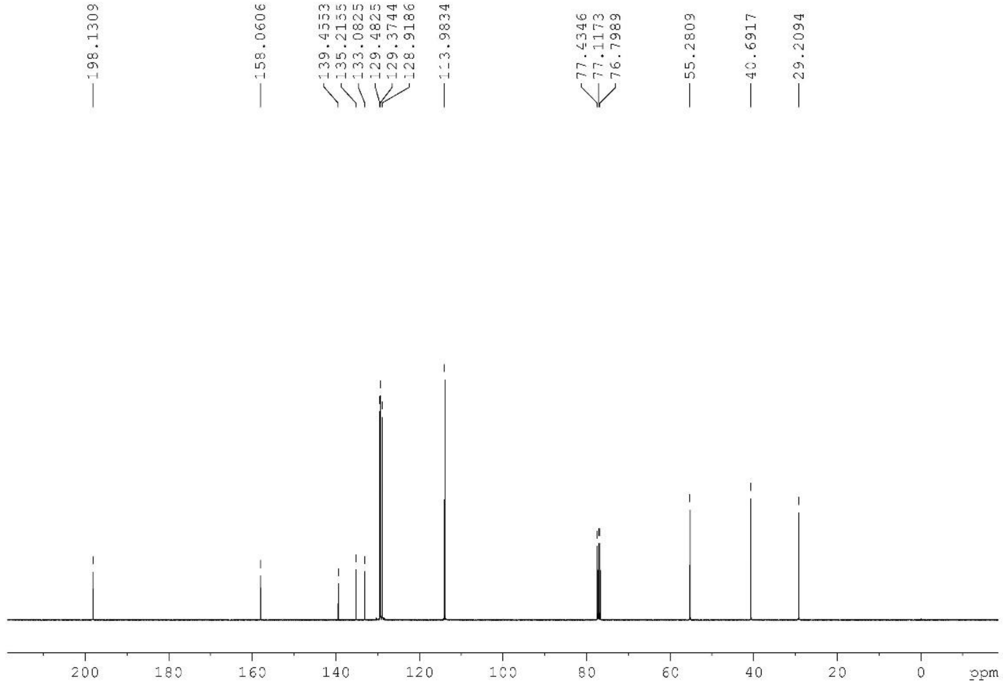

^{13}C NMR spectrum of compound 7fa ($CDCl_3$, 100M)

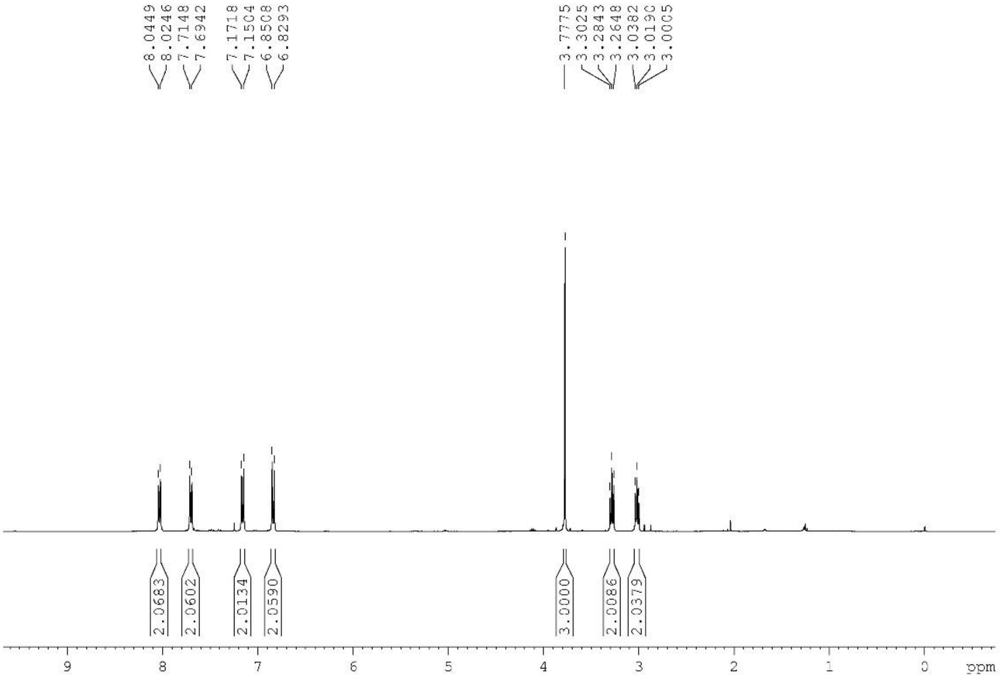

^{1}H NMR spectrum of compound 7ga ($CDCl_3$, 400M)

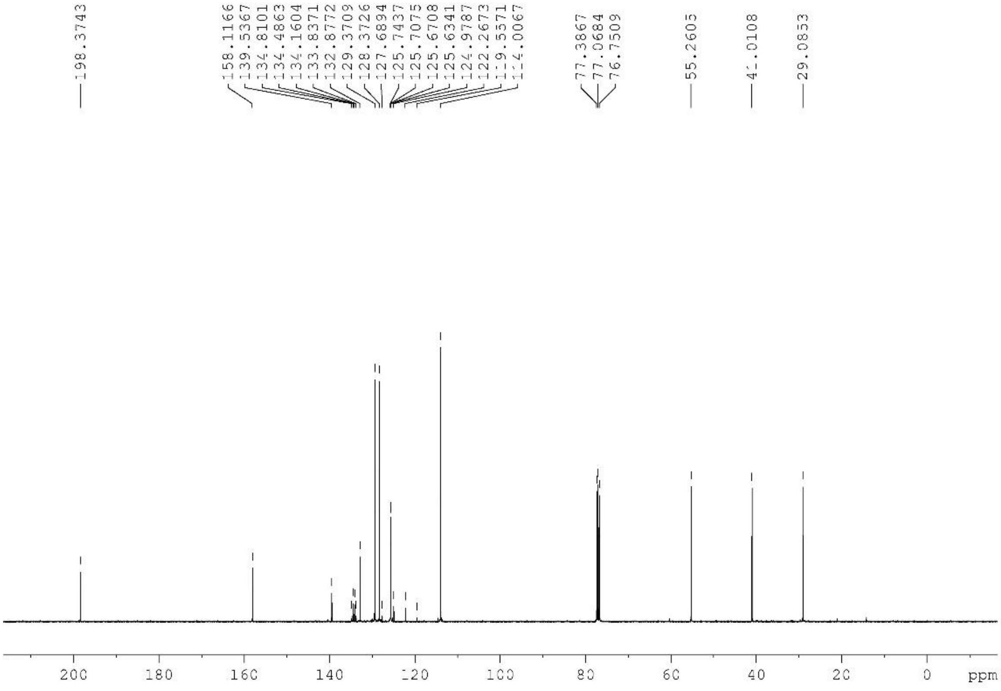

^{13}C NMR spectrum of compound 7ga ($CDCl_3$, 100M)

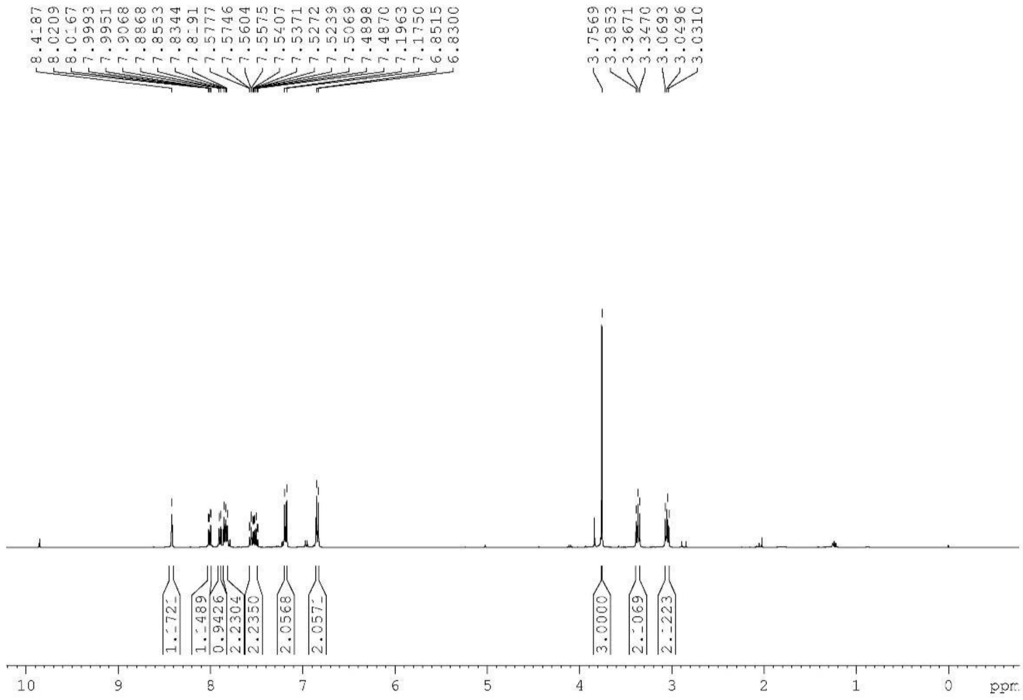

^{1}H NMR spectrum of compound 7ha ($CDCl_3$, 400M)

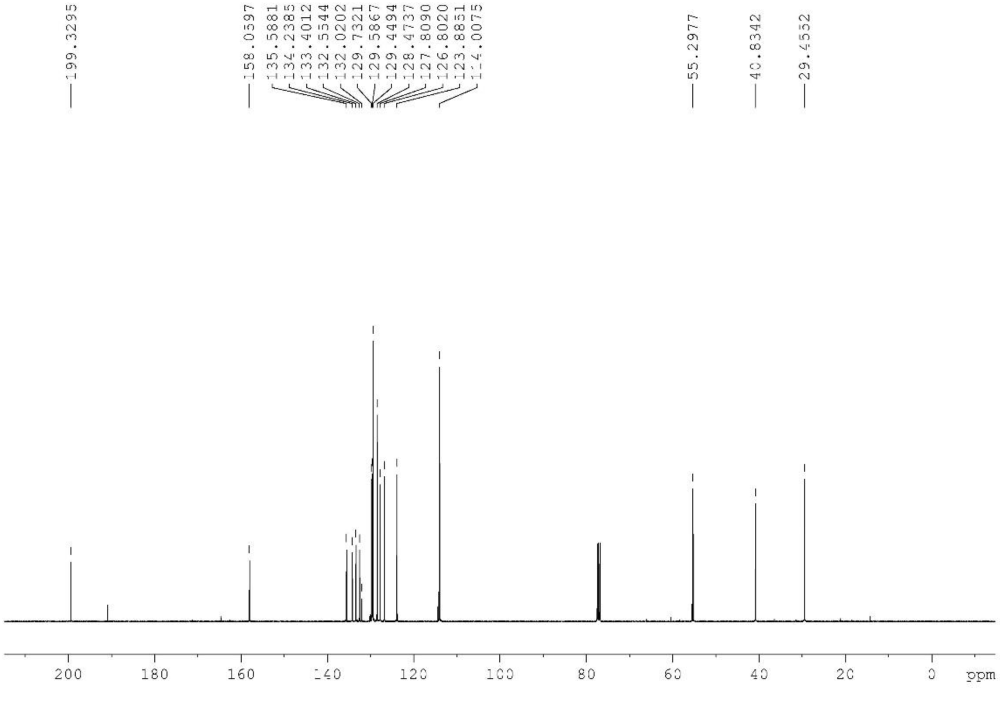

^{13}C NMR spectrum of compound 7ha ($CDCl_3$, 100M)
